# Constellation-based classification of avian reovirus in turkeys reveals shared virus origins among different meat-type farms

**DOI:** 10.3389/fvets.2025.1648247

**Published:** 2025-09-10

**Authors:** Cheng-Shun Hsueh, Michael Zeller, Amro Hashish, Olufemi Fasina, Pablo Piñeyro, Oluwatobiloba Aminu, Mohamed El-Gazzar, Yuko Sato

**Affiliations:** ^1^Department of Veterinary Pathology, College of Veterinary Medicine, Iowa State University, Ames, IA, United States; ^2^Department of Veterinary Diagnostic and Production Animal Medicine, College of Veterinary Medicine, Iowa State University, Ames, IA, United States

**Keywords:** turkey reovirus, genotyping, constellation, avian reovirus, phylogenetic analysis

## Abstract

The US poultry industry suffers significant economic losses due to Avian Reovirus (ARV) infections, which mainly cause arthritis/tenosynovitis in turkeys and chickens. The emergence of outbreaks since 2012 highlights the urgent need for improved epidemiological tools. Given the distinct evolutionary history of each segment of the virus and limited resolution of existing typing methods for ARV based on a single gene, a novel genotyping scheme was developed utilizing a constellation-based genotyping approach to enhance source tracing and control strategies especially for ARV in turkeys. A dataset of 199 ARV sequences from turkey hosts was curated and organized based on branch distances from maximum likelihood phylogenetic trees using TreeCluster. The grouping performance was evaluated and optimized according to established criteria described in this study. The proposed methods selected the M2, S1 σC-encoding region, and L3 genomic segments due to their non-random reassortment and biological significance. The novel scheme identified 8 major genotypes and revealed clear epidemiological links between turkey breeder and meat-type farms, as well as common shared sources among different meat-type farms, suggesting both vertical and horizontal transmission pathways. Additionally, reassortment events were detected using our novel typing scheme, highlighting the complex evolutionary dynamics of ARV. By correlating genotypic patterns with epidemiological data, this study provides a foundation for improved ARV monitoring and disease management.

## 1 Introduction

Avian Reovirus (ARV) is one of the most pressing viral diseases affecting meat-type poultry, contributing to production challenges through arthritis/tenosynovitis, hepatitis, enteritis, and immunosuppression (1). The annual economic impact of ARV is more than $90 million to the broiler industry and over $33 million to the turkey industry (2). However, ARV pathogenesis, accurate diagnosis, virus epidemiology, and control methods are poorly understood.

Currently, ARV characterization can be achieved through genotyping, serotyping, and pathotyping (3, 4). However, none of these typing methods establish a clear link to disease transmission. ARV infection occurs not only horizontally but also through vertical transmission and leads to various clinical presentations; however, ARV is a pantropic virus and the aforementioned clinical presentations are not associated with true pathotypes (5, 6). Additionally, ARV is part of the normal virome of both chickens and turkeys, and multiple viral populations can co-exist and circulate in the same flock (3). Therefore, establishing epidemiological links for viral transmission using sequence typing methods remains challenging (1, 3, 6). Although several ARV sequence typing schemes, based on single (7) or multiple gene (8–10) targets, have been proposed, those have not yet been fully developed and optimized to accurately reflect the epidemiologic relatedness between cases to identify the source of infection. Furthermore, the evolutionary history of ARV in poultry appears to be distinct between turkeys and chickens, as turkey reoviruses are mainly classified into Genotype Cluster 2 under Kant’s σC genotyping scheme (7, 11–13) whereas in chickens, Genotype Clusters 1–7 have been identified in the United States (14), with a subset of these groups (GC I–VII) reported in Asian, African, and European countries with prevalence varying by year (15–17). This classification provides limited resolution for detailed genetic characterization of the turkey population, thereby hindering epidemiological investigations, particularly for the US commercial turkey industry. Therefore, developing a unified genotypic classification method that can utilize genotypic variation to define epidemiology can provide valuable insights into disease transmission, pathogenesis, and diagnosis and enable the formulation of improved prevention and control strategies.

For segmented viruses, because of their capacity for reassortment, genetic characterization and clustering are typically performed on individual segments. These segments are then formed into a constellation to define specific genotypes in relation to host or geographic distribution, as exemplified by influenza viruses and Group A rotavirus (18–22). However, a comprehensive, large-scale analysis of whole viral segment reassortment has yet to be conducted for ARV, leaving uncertainty about which segments are more prone to reassortment and whether that reassortment occurs randomly. This lack of understanding poses a challenge in selecting appropriate anchors for intersegmental comparison.

The elective packaging model of non-random reassortment is widely accepted in segmented virus evolution, as the number of reassortant genotypes observed in both experimental and natural conditions is significantly lower than theoretically expected under the random reassortment model (23). This suggests that interactions between these genes or their protein products are less tolerant of variation between parental strains and may serve a foundational concept for segmented virus genotyping approach (19, 23). An example of this is hemagglutinin (HA) and neuraminidase (NA) genes in highly pathogenic avian influenza viruses, which has a lower frequency of reassortment events, and as a result, has been used as the anchor for genotyping (18, 19, 24). Non-random reassortment has also been observed in *Mammalian orthoreovirus* with certain gene combinations, including L3-S1 and L1-S1 showing a lower frequency of reassortment events, which could be considered into genotyping development (25).

Other genotyping methods typically consider biologically and functionally important factors. In Reoviridae, surface proteins play a crucial role in pathogenicity, host specificity, and immune response (22). Accordingly, for Group A Rotavirus, VP6, VP4, and VP7 genes serve as the primary anchors for genotyping (3, 21). More specifically, Rotavirus VP6 (inner capsid), is highly immunogenic and classifies Rotavirus into five major groups (A–E) (21), while VP4 (spike-like outer capsid protein) and VP7 (outer capsid protein) both elicit neutralizing antibodies (20). Similarly, analogous segments in ARV have been suggested to possess similar biological functions and potential antigenic properties: VP6 corresponds to λC (encoded by L3) in ARV, VP4 to σC (encoded by S1), and VP7 to μB (encoded by M2) (26, 27). Combining these two concepts, we established an ARV genotype classification system incorporating all genome segments, with M2, S1 σC-encoding region, and L3 genes as primary anchors. This approach maximizes biological significance by considering (1) the non-random reassortment evolution model and (2) the critical biological functions of these segments, in combination with the remaining segments to enhance resolution, enabling more precise classification of virus populations at the farm level.

This study focuses on: (1) developing and evaluating the performance of a segment-based genotyping scheme for ARV, particularly in turkey hosts, and (2) correlating this genotyping approach with epidemiological data to trace infection sources at the parent-progeny farm level. This genotyping scheme will be a promising tool for epidemiologic investigations, enhancing disease prevention efforts, addressing the long-standing needs of the poultry industry, poultry clinicians, and veterinary diagnosticians. Additionally, this genotypic scheme can be the basis for other schemes to build on and attempt to classify ARV and predict more complex phenotypic characteristics such as antigenicity (serotypes) and/or pathogenicity (pathogenic vs. non-pathogenic).

## 2 Materials and methods

### 2.1 Collection of sequences and datasets curation

The dataset includes case submissions from the Iowa State University Veterinary Diagnostic Laboratory (ISU-VDL) and records generously provided by two industry collaborators (Breeder Company A and Breeder Company B). A total of 199 whole genome sequences from 181 turkey cases were analyzed: 78 from ISU-VDL, 61 from Breeder Company A, and 42 from Breeder Company B (Supplementary Table S1). Only sequences of each segment covering >90% of the full open reading frame (ORF) were included in the analysis. These sequences were aligned using MAFFT v7.490 in Geneious Prime 2023 (Biomatters) (28). Maximum-likelihood (ML) phylogenetic trees were constructed for each genomic segment using IQ-TREE (29) with automated model selection using ModelFinder (30). The best-fitting nucleotide substitution model was determined based on the Bayesian Information Criterion (BIC) (Supplementary Table S2). Tree topologies were validated via ultrafast bootstrap analysis with 1,000 replicates based on absolute distances (31). A subset of sequences representing each clade for each segment was subsequently selected *ad hoc* and queried against the NCBI Nucleotide database with a limit of 100 target sequences per query. A final 29 to 77 sequences per segment from NCBI Nucleotide database were retained, including ARV sequences from hosts other than turkeys. This data set was compiled after filtering out duplicate sequences and those with >10% missing nucleotides in the full-length ORF from an initial set of 700–1,200 sequences queried from NCBI Nucleotide database. Finally, viral sequences from ISU-VDL, Breeder Company A, Breeder Company B, and GenBank were collated, re-aligned using MAFFT, and analyzed for phylogenetic relationships using Maximum-Likelihood phylogenetics (IQ-TREE) with 1,000 bootstraps. Sequences with accompanying metadata, such as sampling time, location (State), farm type of origin (e.g., breeder or meaty-type growing farms), organ(s) of the viral isolates, and clinical presentation, were retrieved from ISU-VDL and collaborators (Supplementary Table S1). These curated datasets were utilized to develop the genotyping scheme. The naming of the isolate is based on the consensus sequences designed as: species/geographic region/lab identification number/tissue of isolation/year of isolation (14).

### 2.2 RNA extraction and reverse transcription-PCR (RT-PCR)

The samples from submitted flocks were collected as a pool and processed using 2010 Geno/Grinder (SPEX, Meutuchen, NJ) to create homogenates for viral RNA isolation. Viral RNA was extracted from the homogenate using the MagMAX™ Pathogen RNA/DNA kit (ThermoFisher Scientific, Waltham, MA) following the manufacturer’s instruction. The isolated RNA was tested for ARV by published real-time RT-qPCR protocols using primers to detect the conserved region of the M1 genome (32).

### 2.3 Virus isolation and whole genome sequencing (WGS)

Virus isolation was performed following a previously published protocol (11). Freshly homogenized tissues were first centrifuged at 1,200 rpm for 5 min, and the supernatant was passed through a 0.45 μm filter. The filtered solution was then diluted in serum-free media and used to inoculate LMH cells (ATCC Number CRL-2117) cultured in 6-well plates at 37°C under 4–5% CO₂ in a humidified incubator. The cells were monitored for cytopathic effects (CPE) over 7 days. Supernatants from wells exhibiting CPE were collected, subjected to a single passage, and subsequently stored at −80°C. Viral RNA was extracted using the MagMAX™ Pathogen RNA Kit (ThermoFisher Scientific, Waltham, MA) with the KingFisher Flex system. Double-stranded cDNA was then synthesized using the NEXTflex™ Rapid RNA-Seq Kit (Bioo Scientific Corp, Austin, TX) (33). The sequencing library was prepared using the Nextera XT DNA Library Preparation Kit (Illumina, San Diego, CA) with dual indexing. Pooled libraries were sequenced on an Illumina MiSeq platform at the Next-Generation Sequencing (NGS) section of the ISU-VDL using the 600-Cycle v3 Reagent Kit (Illumina) to generate 300 base-pair paired-end reads, following standard Illumina protocols. Raw reads were quality-checked using FastQC (v0.11.9) and processed with Trimmomatic (v0.39) (34). High-quality reads were aligned to reference sequences for each avian reovirus segment from the NCBI database using BWA-MEM (v0.7.17). Reads mapped to reference sequences were extracted with SAMtools (v1.7) (35) and de-novo assembled using ABySS (v2.2.4) (36) and SPAdes (v3.13.0) (37). The final assemblies were validated using BLASTn and manually curated in IGV (v2.16.0) to generate a consensus sequence for each segment (38).

### 2.4 Viral sequence clustering

A phylogenetics-based workflow for clustering ARV sequences of each genome segment was implemented to establish epidemiologic links between flocks. This approach used a tree-based clustering to define monophyletic clades from maximum likelihood trees with the “TreeCluster” package in Python (39). The “Max_clade” method was employed to cluster sequences based on a maximum pairwise distance (t) between leaves within a cluster. Clusters identified in the trees were labeled and numbered sequentially with Arabic numerals starting from 1. Sequences labeled ‘-1’ represent singletons, indicating they did not form a group with the defined distance threshold using the Max_clade method. Before epidemiological analysis, the clustering threshold was selected based on range that approximately represented 10–20 years of viral evolution, as determined by mutation rate (13). After initial testing with distance (t) thresholds ranging from 0.025 to 0.045, thresholds of 0.035 and 0.045 were used for clustering performance evaluation, because a 0.025 threshold resulted in: (1) a high number of singletons, (2) high nucleotide homology within clusters limiting the temporal coverage, and (3) overly granular clustering in phylogenetic trees (Table 1). To evaluate clustering performance and assess the placement of sequences in clusters near the borderline threshold, multidimensional scaling (MDS) was performed using the Python package Scikit-learn (40). Specifically, nucleotide difference pairwise matrices for each segment were generated in Geneious, annotated with assigned cluster numbers, sorted accordingly, and visualized in a low-dimensional space using MDS. MDS clustering quality was further assessed using silhouette score analysis, where scores range from −1.0 to 1.0 (41). Negative values or scores near 0.0 indicate poor cluster separation, scores above 0.5 suggest reasonable clustering, and scores exceeding 0.7 denote optimal clustering performance. Considering the epidemiologic data only available from ISU-VDL and collaborators, if clusters formed by these two sources have a silhouette score below 0.5, our approach involves investigating the underlying causes (e.g., phylogenetic relationships with closely related clusters) and refining the clustering process to achieve scores above 0.5. To better visualize demarcation and pairwise nucleotide identity among clusters defined by TreeCluster, average nucleotide identity percentages between the ORFs of each ARV genome segment were calculated using the pairwise similarity tool in Geneious. The output sequences were annotated with cluster numbers, sorted, and analyzed using Python packages NumPy and Pandas to determine inter-cluster and intra-cluster nucleotide similarity, with results visualized as pairwise heat map matrices.

**Table 1:** Comparison of singleton numbers, lineage numbers, lowest nucleotide homology within clusters, and estimated temporal coverage at phylogenetic distance thresholds of 0.035 and 0.045 using TreeCluster.

Segment	MR (s/s/y)	Length (bp)	Distance threshold 0.025				Distance threshold 0.035				Distance threshold 0.045			
			Singleton#	Lineage#	Nt similarity (%)	Temporal coverage(year)	Singleton#	Lineage#	Nt similarity (%)	Temporal coverage(year)	Singleton#	Lineage#	Nt similarity (%)	Temporal coverage (year)
L1	0.00162	3,882	19	18	97.73	14.00	16	14	97.09	17.94	11	11	95.90	25.28
L2	0.00137	3,780	17	17	97.70	16.83	12	15	96.81	23.34	8	13	96.16	28.09
L3	0.00462	3,858	21	16	97.67	5.04	19	12	96.71	7.12	15	10	96.12	8.39
M1	0.00142	2,199	17	24	97.73	16.04	15	22	96.73	23.11	10	19	95.95	28.62
M2	0.00100	2031	16	10	95.89	41.10	12	10	95.42	45.80	8	9	95.42	45.80
M3	0.00124	1908	19	17	97.69	18.66	13	17	96.80	25.85	8	14	96.02	32.15
σC	0.00245	1,020	26	22	96.23	15.39	19	19	95.89	16.78	17	18	95.81	17.10
S2	0.00136	1,251	20	25	97.84	15.85	17	20	96.88	22.89	12	16	96.24	27.59
S3	0.00163	1,104	20	20	95.18	29.52	17	18	95.18	29.52	12	17	93.58	39.31
S4	0.00117	1,104	21	21	97.46	21.73	12	19	96.71	28.14	8	16	95.50	38.49

MR, mutation rate; s/s/y, substitution/site/year; bp, base pair; #, number; Nt, nucleotide.

### 2.5 Classification criteria

The classification criteria are summarized in Table 2. The genotyping scheme was established by concatenating cluster numbers (Arabic numerals) of each segment for each isolate in the order: M2-σC-L3-L1-L2-M1-M3-S2-S3-S4. For epidemiological analyses, priority was given to M2-σC-L3 constellations to identify major links (e.g., between genetic breeder companies and growing and feeding operations), while the remaining segments provided finer resolution allowing differentiation at the farm or flock level. For clarity, σC gene refers to the σC-encoding region of the S1 gene (42) and is used consistently in the following text, figures, and tables.

**Table 2:** Criteria for classification of ARV isolate.

Criterion	Description
1	Sequence of each segment with nucleotides covering more than 90% of full-length open reading frame
2	Phylogenetic tree topology using Maximum likelihood method with 1,000 bootstrap replicates
3	Assignment of cluster numbers for each segment with Arabic numerals by using TreeCluster with MaxClade method under phylogeny distance threshold of 0.035
4	Concatenating cluster numbers in the order M2-σC-L3-L1-L2-M1-M3-S2-S3-S4
5	Genotypes assigned with Roman numerals created only when constellation patterns of M2-σC-L3 observed in more than four isolate sequences
6	Minor genotype created when (a) constellation patterns of M2-σC-L3 observed in less than four isolate sequences, (b) missing or unclassified sequences (singletons) in more than one segment of M2-σC-L3, and (c) missing or unclassified sequences (singletons) in more than four segments of L1-L2-M1-M3-S2-S3-S4
7	Assignment of a sub-genotype using numerical-decimal address (Roman-Arabic numerals separated by periods, e.g., I.1 and I.2) when a distinct epidemiologic link (i.e., source of virus) identified within a M2-σC-L3 constellation pattern but having different L1-L2-M1-M3-S2-S3-S4 constellation from the original genotype
8	Assignment of a new genotype with a Roman numeral when newly identified virus diversity (group of virus previously placed in minor genotype or undescribed) meeting all criteria

The frequency of unique constellation patterns was calculated using the Python package Pandas. Constellation patterns of M2-σC-L3 observed in fewer than 4 of 199 sequences (2%) were classified as minor genotype. Isolates with missing or unclassified sequences (singletons) in (a) more than one segment of M2, σC, and L3, or (b) in more than four of the remaining seven segments, were also categorized as minor genotype and excluded from epidemiologic link analysis. Genotypes (Roman numerals) were assigned to distinct constellation patterns of M2-σC-L3 segments. The phylogenetic tree with whole-genome cluster constellations was visualized using FigTree v1.4.4^1^ and Interactive Tree of Life (iTOL v6) (43).

## 3 Results

### 3.1 Comparison of clustering performance at phylogenetic distance thresholds of 0.035 and 0.045 using TreeCluster

#### 3.1.1 Multidimensional scaling (MDS) and silhouette score analysis

**Figure 1:**
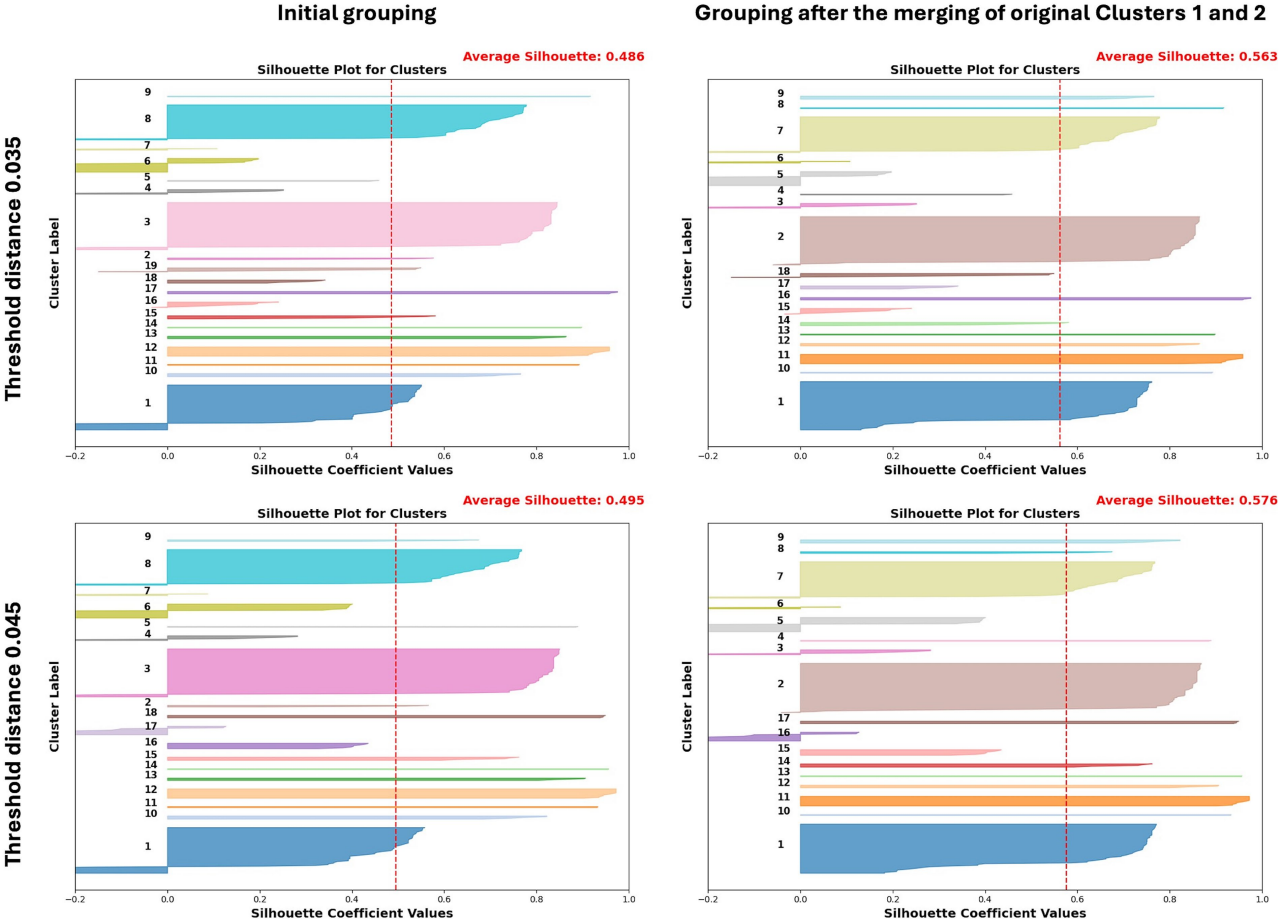
Silhouette score comparison of sequence distances before and after merging Clusters 1 and 2 in σC clustering, using TreeCluster under distance thresholds of 0.035 and 0.045.

Following cluster assignment using TreeCluster with distance thresholds of 0.035 and 0.045, we identified 10–22 clusters at the 0.035 threshold and 9–19 clusters at the 0.045 threshold, depending on the segments (Table 1). Additionally, there were 12–19 singletons (4.7–8.0%) at the 0.035 threshold and 8–17 singletons (3.1–4.7%) at the 0.045 threshold for each cluster (Table 1). To assess whether the genotypic clustering from the phylogenetic tree assigned by TreeCluster is recapitulated with nucleotide similarity levels, we computed the Euclidean distances of the full ORF genome from multiple sequence alignments of the ARV to seek support for the genotypic clustering obtained by TreeCluster. The initial MDS and silhouette score analysis revealed an overall optimal clustering distribution under the thresholds of both 0.035 and 0.045 with a few to some segments showing clusters that have a silhouette score lower than 0.5 (indicating weak clustering) (Supplementary Figures. S1–S3). These clusters with score lower than 0.5 mainly consist of sequences from GenBank, which were uploaded sporadically, often as single cases or small-scale sequence analyses conducted some time ago versus the large-scale dataset collected over the past 5 years in our study. In the σC segment, initial clustering resulted in 19 and 18 clusters for 0.035 and 0.045 distance threshold, respectively (Table 1). However, Cluster 1, which includes a large sequence set from ISU-VDL and collaborators, exhibited a low silhouette score (0.33 for distance of 0.035 and 0.34 for distance of 0.045) (Figure 1). Analysis revealed that Cluster 2 is genetically and phylogenetically similar to Cluster 1 with a nucleotide similarity of 96.94%. This similarity falls within the borderline cutoff values for tree distances of 0.035 and 0.045, which likely contributed to the poor demarcation of the clusters. As a solution to this, Clusters 1 and 2 were manually merged into a single Cluster 1 that significantly improved the silhouette scores from 0.33 to 0.61 for distances of 0.035, and from 0.34 to 0.63 for distances of 0.045 (Figure 1). Following the merging of Clusters 1 and 2 into a single Cluster 1, the original Cluster 3 was reassigned as Cluster 2, Cluster 4 was reassigned as Cluster 3, and so on.

When comparing all 10 segments with different distance threshold, clustering under the 0.035 threshold yielded more segments with silhouette scoring > 0.7, while only 3 segments (L1, M1, and σC) exhibited a higher silhouette scoring at the 0.045 threshold compared to the 0.035 threshold (Table 3). Additionally, individual clusters with silhouette scores higher than 0.7 (indicating strong clustering) were more frequently observed at the 0.035 threshold (82 out of 165 clusters, 49.7%) compared to the 0.045 distance threshold in the overall clusters (53 out of 132 clusters, 40.1%) (Table 3), suggesting a better clustering performance at the 0.035 threshold, based on objective silhouette scores using nucleotide similarity as a reference.

**Table 3:** Comparison of clustering performance at phylogenetic distance thresholds of 0.035 and 0.045 using TreeCluster.

		Distance threshold	
		0.035	0.045
Number of clusters with silhouette scoring > 0.7		82	53
Total cluster number		165	132
Percentage (%) of clusters with silhouette scoring > 0.7		49.7	40.1
Silhouette Coefficient	L1	0.73	0.74
	L2	0.77	0.66
	L3	0.79	0.74
	M1	0.62	0.63
	M2	0.77	0.76
	M3	0.69	0.60
	σC	0.56	0.58
	S2	0.69	0.64
	S3	0.62	0.61
	S4	0.60	0.53

#### 3.1.2 Viral isolate coverage rate analysis based on M2-σC-L3 constellation patterns

**Figure 2:**
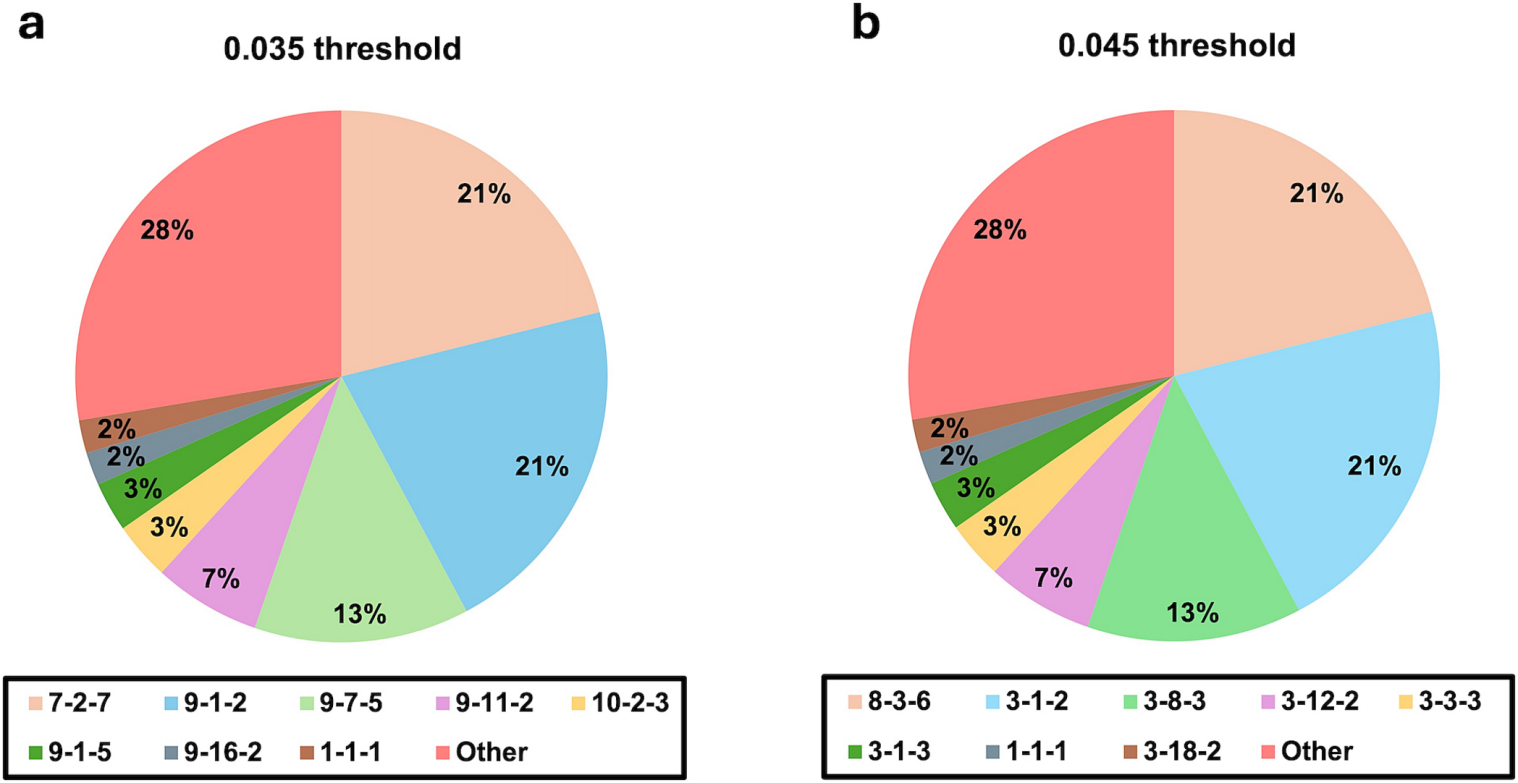
Comparison of sequence coverage under distance thresholds of **(a)** 0.035 and **(b)** 0.045 based on M2-σC-L3 constellation patterns.

When applying the criterion of considering constellation patterns of M2-σC-L3 observed in more than 4 of 199 cases (2%), both thresholds yielded 8 distinct constellation patterns, covering 144 cases (72.4%) (Figure 2), indicating no difference in the performance of viral isolate coverage between the 0.035 and 0.045 thresholds. Additionally, all these clusters within 8 major constellation patterns had silhouette scores above 0.6.

Based on the clustering performance analysis, the 0.035 threshold was selected as it provides a balance between maintaining distinct clusters with higher cluster numbers and achieving a silhouette score above 0.7. Additionally, this clustering method ensures appropriate case coverage with no difference from 0.045 threshold and is therefore subject to test for its effectiveness in establishing genotyping and epidemiological links.

### 3.2 M2, σC and L3 as the anchor in constellation genotyping

#### 3.2.1 M2

**Figure 3:**
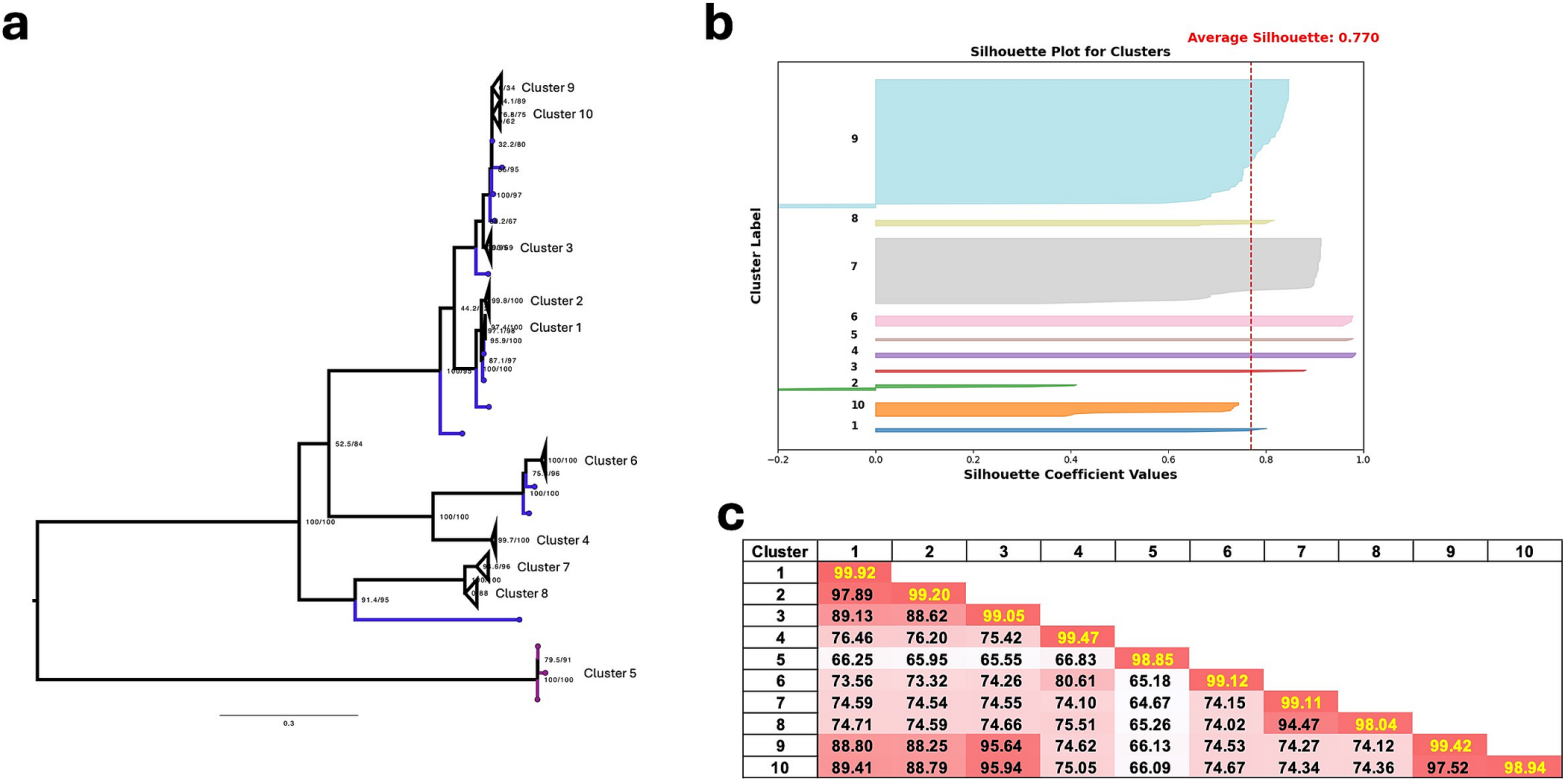
**(a)** Maximum-likelihood phylogenetic tree of the M2 gene. Branches colored in blue indicate singletons. **(b)** Silhouette plot for M2 clusters showing optimal clustering. Negative scores are mostly from GenBank sequences. **(c)** Heat map of average pairwise nucleotide similarity within (highlighted) and between clusters.

A total of 236 sequences were analyzed and assigned to 10 clusters. Most sequences (174/236, 73.7%) were grouped into clusters 7 (*n* = 59) and 9 (*n* = 115). Clusters 3, 4, and 8 consisted of sequences from GenBank, while the remaining clusters (1, 2, 5–7, 9, 10) contained sequences from ISU-VDL and both collaborators. Intra-cluster nucleotide homology was higher than inter-cluster nucleotide homology, revealing an average 99.1% nucleotide identity within clusters and an average 77.3% nucleotide difference between clusters (Figure 3). This finding corresponded to a clear demarcation of clusters in the phylogenetic tree (Figure 3). Inter-cluster nucleotide identity analysis revealed the lowest nucleotide similarity between clusters 5 and 7 (64.7%). Notably, M2 sequences in Cluster 5 are the most divergent from the other cluster groups and are closely related to the Hungarian pheasant reovirus (Reo/HUN/Pheasant/216/2015, 100% identity) and the Hungarian chicken reovirus (924-Bi-05, 95.2% identity) (44, 45). These sequences likely originated from reassortment events involving different geographic regions and unknown vectors, followed by transmission to turkeys in the U.S.

#### 3.2.2 σC

**Figure 4:**
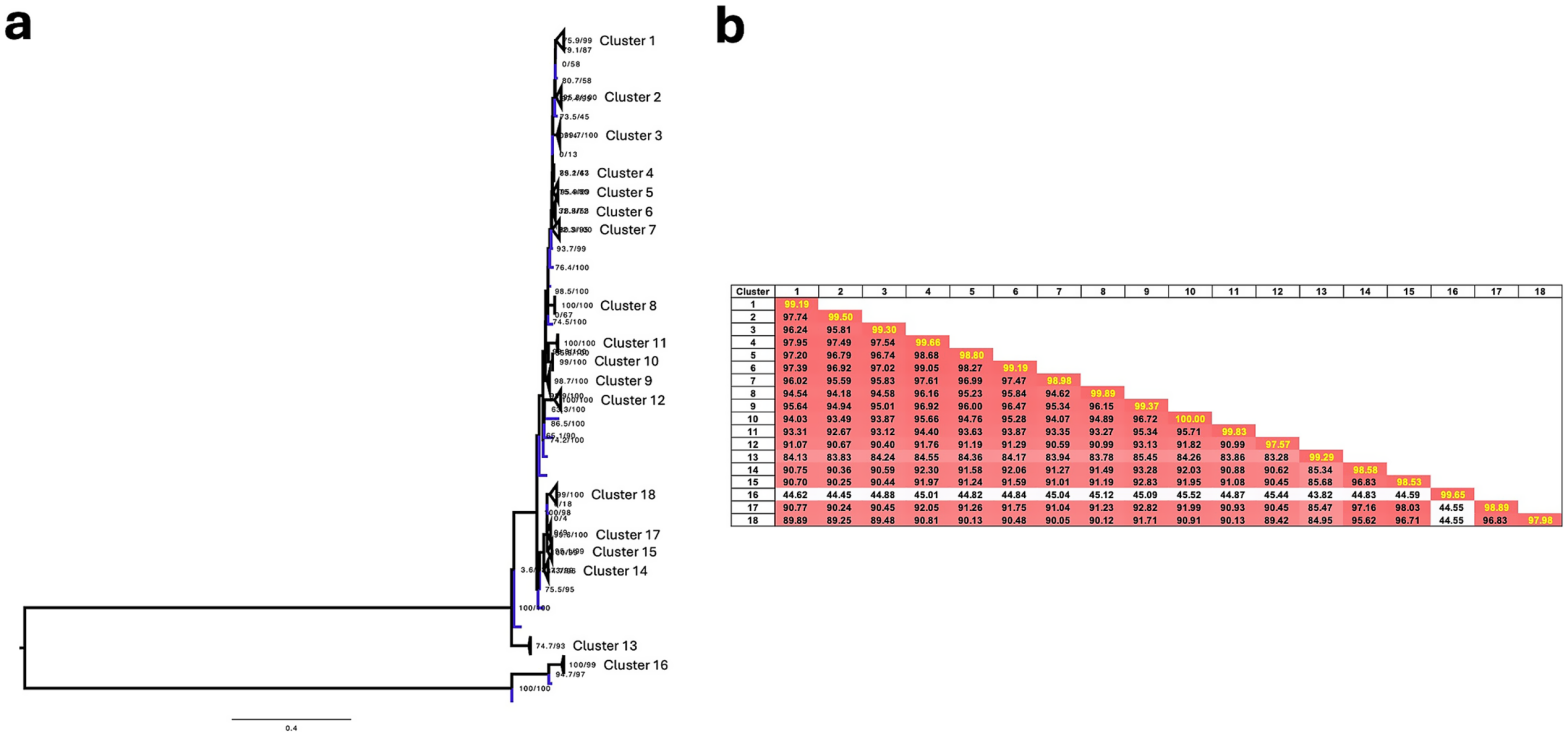
**(a)** Maximum-likelihood phylogenetic tree of the σC gene. Branches colored in blue indicate singletons. **(b)** Heat map of average pairwise nucleotide similarity within (highlighted) and between clusters.

A total of 279 sequences were analyzed in the genotyping study and grouped into 19 clusters. The majority of sequences (173/279, 62.0%) were assigned to clusters 1 (*n* = 63), 2 (*n* = 63), and 7 (*n* = 47), all of which included sequences from ISU-VDL, collaborators, and GenBank. Clusters 3, 4, 5, 6, 8, 9, 10, and 13 contained sequences exclusively from GenBank, 6 of which had fewer than 4 sequences. The clustering method demonstrates an average intra-cluster nucleotide similarity of 99.1% and an average inter-cluster nucleotide similarity of 87.1% that well delineates phylogenetic relationships (Figure 4). The lowest inter-cluster similarity was found between cluster 16 and all other clusters (43.8–45.2%). Sequences in Cluster 16 (n = 4) were phylogenetically distinct, with the highest nucleotide similarity (94%) to NC/SEP-R44/03, a strain isolated in 2003 that caused severe immunosuppression in turkeys (46). Notably, no similar sequences have been reported since its discovery until recently identified in our study.

#### 3.2.3 L3

**Figure 5:**
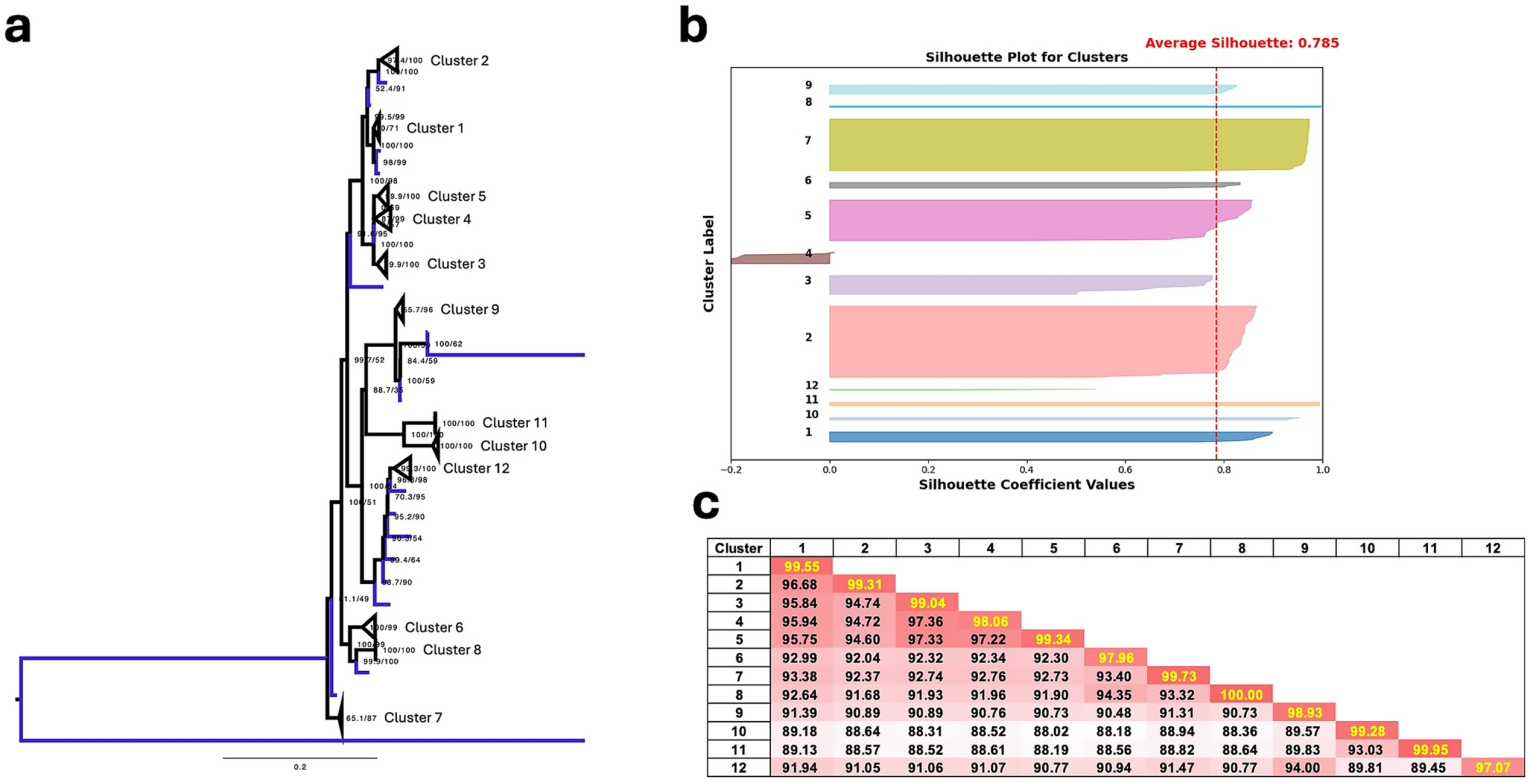
**(a)** Maximum-likelihood phylogenetic tree of the L3 gene. Branches colored in blue indicate singletons. **(b)** Silhouette plot for L3 clusters showing optimal clustering, with negative scores mostly from GenBank sequences. **(c)** Heat map of average pairwise nucleotide similarity within (highlighted) and between clusters.

A total of 236 sequences were analyzed and grouped into 12 clusters. The main clusters (152/236, 64.4%), Cluster 2 (*n* = 66), Cluster 7 (*n* = 48), and Cluster 5 (*n* = 38) were mainly composed of sequences from ISU-VDL and collaborators. Clusters 9 to 12 exclusively comprised sequences from GenBank. The clustering method demonstrates an average intra-cluster nucleotide similarity of 99.0% and an average inter-cluster nucleotide similarity of 90.6% (Figure 5). Clusters 10 and 11 were distinct from the other clusters, sharing 87–92% similarity with the rest. Cluster 10 comprises isolates from 2011 and 2013, including Turkey/USA/MN/2013/TARV-MN11, Turkey/USA/MN/2013/TARV-MN2, and Turkey/USA/MN/2011/TARV-MN4, which were identified during the early 2010s outbreak of turkey reovirus (10). Cluster 11 consists exclusively of Hungarian turkey reoviruses, including Reo/Turkey/HUN188/2016, Reo/Turkey/HUN189/2016, Reo/Turkey/HUN190/2016, and Reo/Turkey/HUN194/2016 (12). Notably, both clusters contain only sequences retrieved from GenBank, with no ISU-VDL submissions.

#### 3.2.4 Other segments

The total sequence and cluster numbers for each segment with intra-cluster and inter-cluster nucleotide similarities and phylogenetic trees are provided in the Supplementary Figures. S4 – S9. The clustering method, based on the nucleotide similarity within the same clusters, provides a temporal coverage of at least 20 years. The proposed clustering method for these segments had optimal visualization of phylogenetic relationships, and these segments are used as a differential tool to offer a higher resolution for source identification at the farm level.

### 3.3 Genotypes of M2-σC-L3 reflect links between breeder farms and meat-type farms

We concatenated the M2, σC, and L3 segments to identify identical constellation patterns, assigning a genotype (Roman numerals) to each pattern. Eight genotypes (I-VIII) were assigned, and the metadata, including farm types, bird source, collection dates, location, and clinical presentations, are summarized in Table 4 and detailed in Supplementary Table S1.

**Table 4:** Summary of sample collection timing, farm type, state, bird source, and sampling purpose for major genotypes.

Genotype	Constellation^a^	Year	Farm type	State	Bird source	Sampling purpose	Lesion
I.1	7-2-7^b^	Late 2022-Early 2023	Meat-type farm	IA, MN	Breeder Company A	Surveillance	NA
		Mid 2023	Mixed operation	NC	Breeder Company A + B	Diagnostic	Necrotizing hepatitis
					Breeder Company B	Surveillance	NA
I.2	7-2-7^c^	2024	Meat-type farm	IA, MN	Breeder Company B	Surveillance	NA
						Diagnostic	Necrotizing hepatitis
II	9-1-2	2023	Breeder farm	MO	Breeder Company B	Surveillance	NA
			Meat-type farm	IA, MN, PA	Breeder Company B	Surveillance	NA
						Diagnostic	Tenosynovitis; Necrotizing hepatitis
III	9-7-5	2023	Breeder farm	NA	Breeder Company A	Surveillance	NA
		2020–2023	Meat-type farm	NA	Breeder Company A	Surveillance	NA
IV	9-11-2	2023–2024	Meat-type farm	MN	Breeder Company B	Surveillance	NA
			Mixed operation	IN, OH	Breeder Company A + B	Diagnostic	Tenosynovitis
V	10-2-3	2022–2023	Meat-type farm	IA	Breeder Company A	Surveillance	NA
						Diagnostic	Tenosynovitis; Necrotizing hepatitis
VI	9-1-5	2022–2023	Breeder farm	NA	Breeder Company A	Surveillance	NA
VII	1-1-1	2020	Mixed operation	NA	Breeder Company A + B	Surveillance	NA
VIII	9-16-2	2023	Meat-type farm	IA, MN	Breeder Company B	Surveillance	NA

^a^, M2–σC–L3; ^b^, M2 [7]–σC [2]–L3[7]–L1[8]–L2[2]–M1[10]–M3[15]–S2[17]–S3[12]–S4[2]; ^c^, M2[7]–σC[2]–L3[7]–L1[8]–L2[9]–M1[5]–M3[15]–S2[18]–S3[12]–S4[5]; NA, not applicable; IA, Iowa; MN, Minnesota; NC, North Carolina; MO, Missouri; PA, Pennsylvania; IN, Indiana; OH, Ohio.

#### 3.3.1 Genotype I (M2 [7] – σC [2] – L3 [7])

##### 3.3.1.1 Genotype I.1 (M2[7] – σC[2] – L3[7] – L1[8] – L2[2] – M1[10] – M3[15] – S2[17] – S3[12] – S4[2])

**Figure 6:**
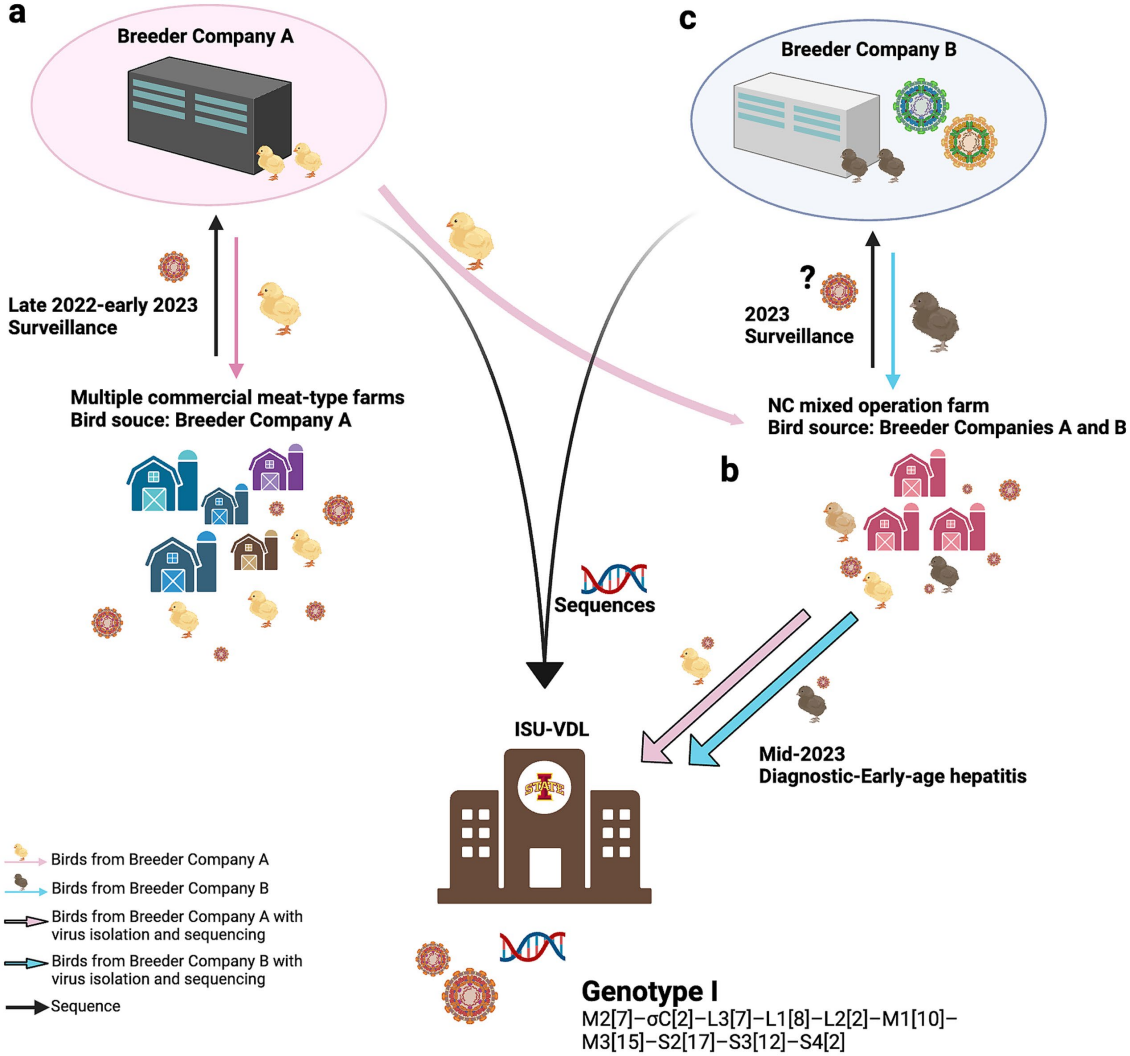
Case scenario of Genotype I.1 (M2[7]–σC[2]–L3[7]–L1[8]–L2[2]–M1[10]–M3[15]–S2[17]–S3[12]–S4[2]). The identification of this virus population began with Breeder Company A, which detected it in various meat-type farms sourcing birds exclusively from Company A during 2022–2023 **(a)**. In 2023, a group of viruses was isolated from a mixed-operation farm in North Carolina (NC) that sourced birds from both Breeder Companies A and B. This NC mixed-operation farm submitted cases to the Iowa State University Veterinary Diagnostic Laboratory (ISU-VDL) in 2023 for general diagnostics, where the virus was isolated, and these cases were diagnosed as young-age hepatitis **(b)**. Subsequent virus isolation and sequencing conducted by Breeder Company B revealed a genomic composition distinct from its internal dataset **(C)**. Using our constellation-based genotyping approach, we determined that all isolates shared the same constellation and were classified as Genotype I.1. The figure was generated using BioRender software (https://www.biorender.com).

The case scenario of this genotype was summarized in Figure 6. Between late 2022 and early 2023, Breeder Company A initiated surveillance across several commercial meat-type farms in different regions, as these farms sourced their birds from this breeder. The surveillance results identified the same genomic constellation among virus isolates from these commercial farms, strongly suggesting a common source for the virus population (Figure 6a).

By mid-2023, several cases of ARV-associated hepatitis were diagnosed in young poults at a mixed-operation farm in North Carolina (NC). Unlike the previously surveyed commercial farms, this NC farm sourced birds from both Breeder Companies A and B, as well as its own stock. Despite differences in location and operation, the virus population at the NC farm exhibited the same genomic constellation as those found in commercial farms only sourcing birds from Breeder Company A, suggesting that Breeder Company A was the common viral source (Figure 6b).

This finding was further supported through comparing sequences from Breeder Company B. The isolates from the NC farm did not match Breeder Company B’s internal ARV sequence database (private dataset) (Figure 6c). This suggest the virus did not originate from Breeder Company B, and it also supports the findings that the virus was likely introduced from Breeder Company A.

Based on the timeline, transmission dynamics, and clinical presentation of early-age hepatitis, this virus population most likely originated from a common source, likely Breeder Company A, and was transmitted vertically to multiple commercial meat-type farms and the NC mixed-operation farm. From there, on the NC farm, the virus spread further, infecting birds from Breeder Company B through horizontal transmission. Although experimental studies suggest a low rate of egg transmission, vertically infected chicks from vertical transmission are considered the primary nucleus of infection, spreading the virus horizontally to the rest of the flock through the fecal-oral route (47, 48). This could explain the observed transmission dynamics of genotype I in the NC farm.

##### 3.3.1.2 Genotype I.2 (M2[7] – σC[2] – L3[7] – L1[8] – L2[9] – M1[5] – M3[15] – S2[18] – S3[12] – S4[5])

In this constellation pattern, the virus was isolated from multiple commercial meat-type farms, all of which sourced birds from Breeder Company B. These samples were collected in early 2024. The majority were surveillance samples with limited information regarding their history or associated lesions, while only one sample, submitted for pathological evaluation, revealed early-age hepatitis, suggesting likely vertical transmission.

#### 3.3.2 Genotype II (M2 [9] – σC [1] – L3 [2])

Genotype II demonstrated clear epidemiological links between breeder and meat-type farms. Initially, this genotype includes ARV isolates exclusively from parent birds within Breeder Company B, collected between 2022 and 2023 by Breeder Company B. In 2023, between March to December, several birds from different meat-type farms were submitted to the ISU-VDL for ARV diagnosis and surveillance. Virus isolates from these farms were grouped into Genotype II with diagnoses of hepatitis and tenosynovitis. Upon reviewing the metadata, all these meat-type farms were found to share the same bird source, Breeder Company B, suggesting evidence of vertical transmission from parents to progenies. Interestingly, few virus sequences from meat-type farms sourcing birds from Breeder Company A were also classified within this genotype. It remains unclear whether these farms had previously acquired birds from Breeder Company B, leading to environmental persistence, or if the virus was introduced through horizontal transmission, such as contact with other commercial farms via personnel, feed trucks, or shared equipment. Notably, no virus sequences directly originating from parent birds of Breeder Company A were classified within this genotype.

#### 3.3.3 Genotype III (M2 [9] – σC [7] – L3 [5])

Genotype III exhibited distinct epidemiological connections between breeder and meat-type farms. Viruses of this genotype were isolated from both grandparent and parent birds within Breeder Company A as part of surveillance. Besides, multiple meat-type farms that sourced offspring from Breeder Company A also had virus isolates classified under this genotype, suggesting vertical transmission events. Since all virus samples were collected for surveillance purposes, the presence of clinical signs in the affected birds is unknown.

#### 3.3.4 Genotype IV (M2 [9] – σC [11] – L3 [2])

Genotype IV includes viruses isolated from birds across multiple meat-type farms that shared the same bird source, Breeder Company B, as well as a mixed operation in Indiana and Ohio, which sourced birds from both Breeder Companies A and B as well as its own stock. Submissions from the mixed operation in Indiana and Ohio revealed lesions of tenosynovitis. Due to the absence of viral sequences directly from parent flocks, a definitive link between the breeders and meat-type farms for this genotype could not be established. Nevertheless, its presence in multiple meat-type farms sharing the same bird source suggests a common origin and/or a specific shared epidemiological event for the virus spread.

#### 3.3.5 Genotypes V–VIII

Genotypes V–VIII suggest that ARV infections across different meat-type farms likely share a common source, originating from either Breeder Company A or Breeder Company B, or potentially from another specific epidemiological event. Genotype V (M2 [10] – σC [2] – L3 [3]) comprises isolates from multiple meat-type farms with all sourcing birds from Breeder Company A with diagnoses of tenosynovitis or hepatitis. Genotype VI (M2 [9] – σC [1] – L3 [5]) includes surveillance samples from grandparent and parent birds from Breeder Company A, consistent with vertical transmission of the virus. Genotype VII (M2 [1] – σC [1] – L3 [1]) consists of isolates from a mixed-operation farm in Indiana and Ohio for surveillance. Finally, Genotype VIII (M2 [9] – σC [16] – L3 [2]) includes isolates from meat-type farms that shared the same bird source, Breeder Company B, and were associated with early-aged hepatitis, likely suggesting vertical transmission.

### 3.4 Identification of reassortments

**Figure 7:**
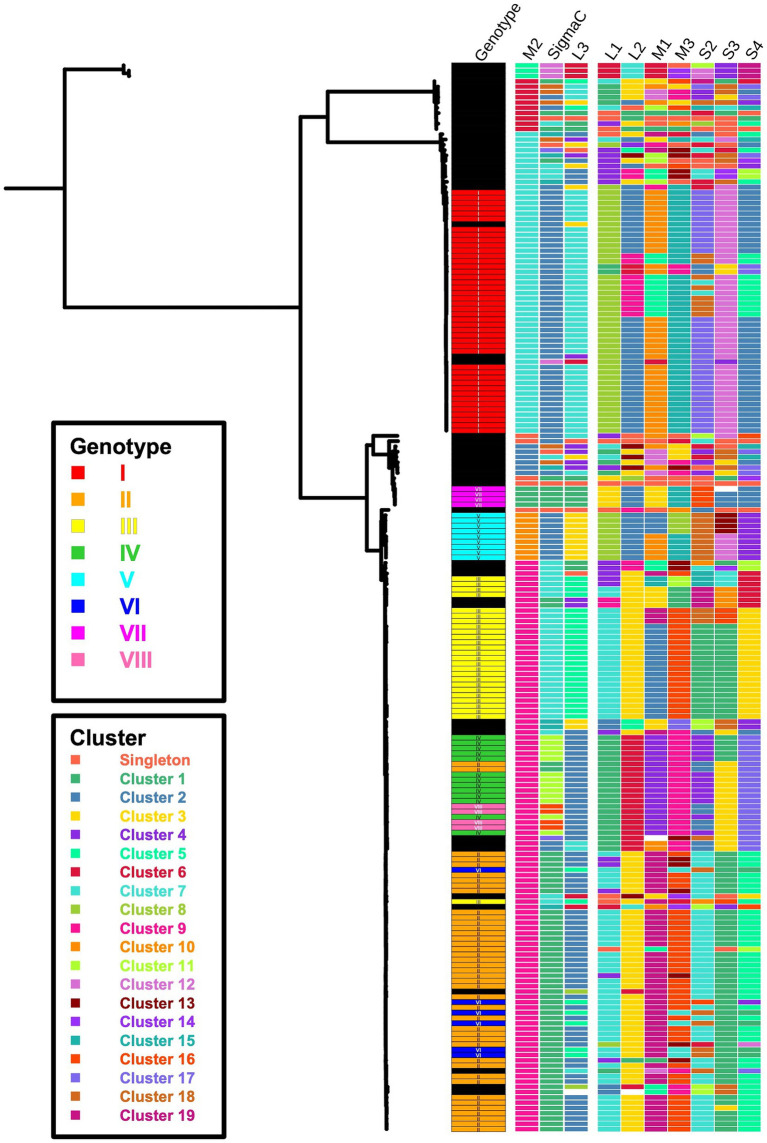
Maximum-likelihood tree of the M2 gene with a color-coded genotype and segment cluster map. The left panel shows the ARV M2 phylogenetic tree. The right panel presents a heatmap of genotype and cluster groups. Black blocks indicate ungrouped (minor) genotypes, and blank spaces represent missing data.

In addition to identifying genotypes, the segment-based approach for sequence classification enables the comparison of genomic data at the segment level between strains or genotypes. This approach supports the common observation that reassortment is a key feature in the evolution of segmented viruses (45, 49, 50). For instance, within Genotype I, two distinct constellation patterns were identified, with 6 segments (M2, σC, L3, L1, M3, S3) remaining consistent, while the remaining four segments (L2, M1, S2, S4) clustered differently (Figure 7). A similar pattern was observed between Genotypes III and IV – both shared the same cluster numbers for segments M2, L3, L1, L2, S2, and S3, but differed in σC, M1, M3, and S4. However, due to the limited number of turkey reovirus isolates and the lack of detailed epidemiologic data available on GenBank, inferring the evolutionary history of genetic reassortment in this study was challenging. Nonetheless, the examples presented here highlight the potential utility of a classification system encompassing all 10 ARV gene segments for studying genetic reassortment in conjunction with epidemiologic links.

### 3.5 Comparison of the genotyping scheme with existing ARV genotyping method

**Figure 8:**
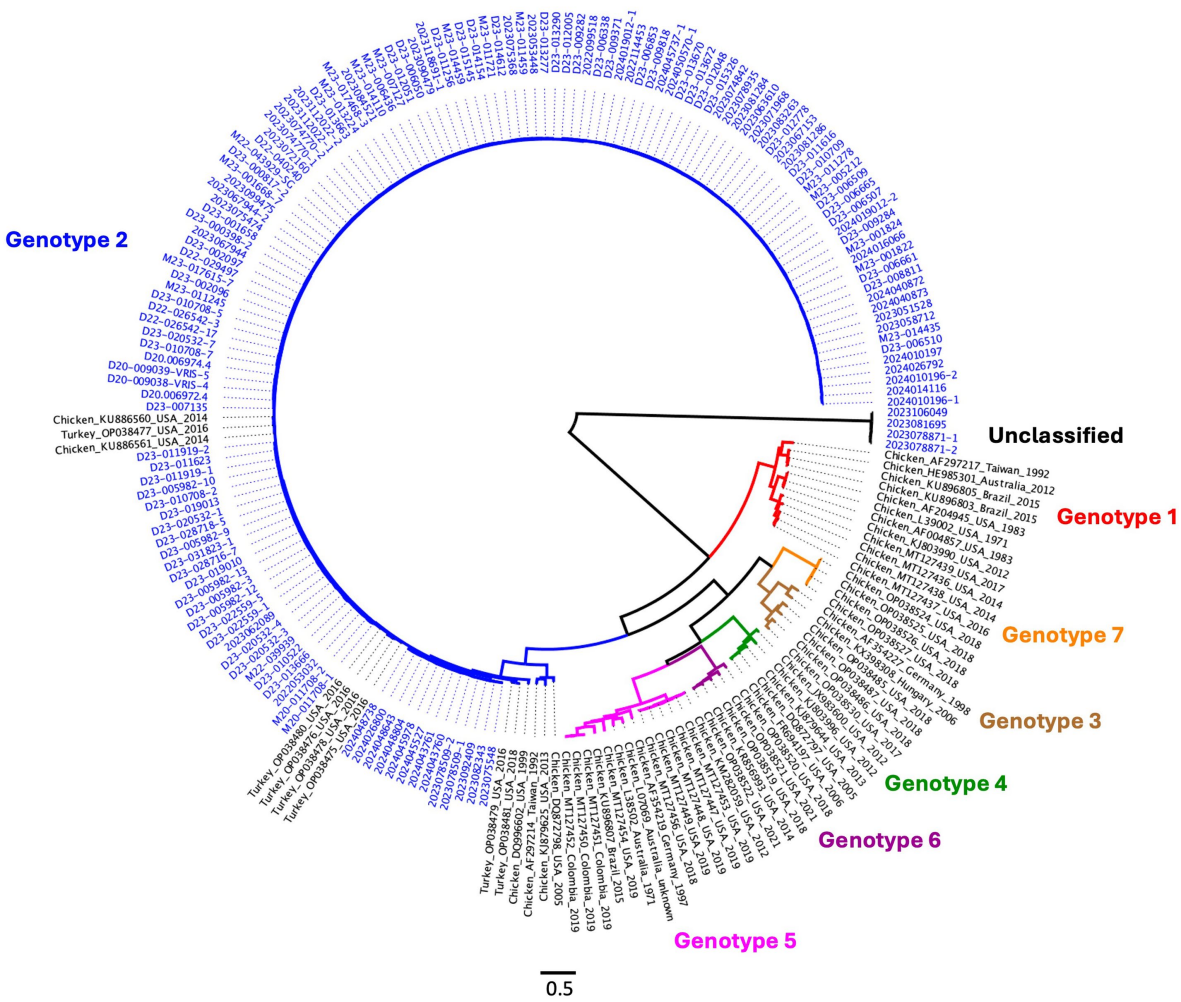
Maximum-likelihood phylogenetic tree based on σC‑encoding S1 gene sequences with 1,000 bootstrap replicates. Self-sampled sequences are highlighted in blue, while the remaining sequences represent reference strains from Egaña‑Labrin et al. (26) and Sellers (14). Most sequences in this analysis were grouped within Genotype Cluster 2 and were phylogenetically related to other turkey‑derived sequences, while a group of viral populations could not be classified using Kant’s scheme. The scale bar indicates nucleotide substitutions per site.

Genotyping using Kant’s method (7, 14, 26) was performed for isolates that could be classified with the proposed typing scheme, and a comparison of both schemes is presented in Figure 8 and Supplementary Table S1. Phylogenetic analysis and genotyping demonstrated that all turkey ARV isolates, regardless of genotype, clustered within Genotype Cluster 2, while an additional group of viral populations could not be classified using Kant’s scheme.

## 4 Discussion

There is a growing need for an Avian Reovirus genotyping scheme that correlates with epidemiological, serological, and pathological classification. The current classification system based on the σC amino acid sequence lacks sufficient resolution for understanding ARV diversity, particularly in turkeys, due to the nature of the virus to reassort and the limited ability of existing typing schemes. In this study, we implemented a genotypic classification system based on phylogenetic clustering approaches on a dataset of turkey reovirus sequences collected from the ISU-VDL database, industrial collaborators, and GenBank. We applied this typing scheme in an epidemiological context to investigate the relatedness of virus populations, with specific focus on the parent-progeny farms to better understand vertical transmission events, the most likely source of infection. Furthermore, the constellation of the cluster number from multiple segments offers an advantage over single-segment typing or the traditional Kant’s typing scheme by providing a more comprehensive framework for linking epidemiological connections with constellation patterns and detecting segment reassortments. Based on this, this typing scheme will allow for creating more robust population structure, which are crucial for understanding viral evolution and transmission dynamics.

Sample collection and molecular typing conducted by diagnostic labs and animal health professionals are primarily for monitoring and studying pathogen transmission dynamics. However, ensuring high-quality samples and sequences is a fundamental and critical first step in such applied research, as poor-quality data can introduce significant biases. As with studies on other segmented RNA viruses, such as avian influenza virus, a 90% sequence length cutoff is widely accepted as a quality threshold for genetic, phylogenetic, and genotyping analyses (19, 51, 52). In this study, the classification of S1 is based on the σC-encoding region, which is the only structural protein encoded by this tricistronic segment. Due to its immunogenic properties and the abundance of publicly available sequence data, establishing a genotyping approach centered on the σC-encoding region would be advantageous for future research, particularly for investigating associations between genotypic variation and immunogenic characteristics.

In this study, we employed Illumina sequencing technology. Among the turkey ARV sequences available in GenBank, the majority have likewise been generated using either the Sanger or Illumina platforms (53). Both methodologies differ substantially in their underlying principles, throughput capacity, and resolution. Sanger sequencing yields longer, highly accurate reads, and is well suited for targeted sequencing of specific genomic regions (54). In contrast, Illumina sequencing is a high-throughput approach that produces shorter reads, deep coverage, and increased sensitivity for the detection of minor variants (53). For the present study, Illumina sequencing was selected to enable comprehensive analysis of all ARV genome segments. Nonetheless, Illumina data require rigorous quality control, assembly, and error correction due to increased susceptibility to certain sequencing errors. Consequently, when comparing newly generated Illumina sequences to those deposited in GenBank, it is important to consider these methodological differences as potential contributors to observed sequence variation.

We obtained sequences from NCBI GenBank to define overall global ARV genetic variation in turkeys and reconstruct phylogeny alongside sequences from ISU-VDL and collaborators. Therefore, one of the limitations of this study is sampling bias, as the majority of sequences analyzed (correlating virus source with available epidemiologic data) originated from the only two turkey breeder companies in the United States and a single diagnostic laboratory. Although this dataset provides valuable insights into the genetic diversity of turkey avian reoviruses, the limited source diversity may not fully reflect the breadth of viral populations circulating in other geographic regions (e.g., Europe or Asia) or across different production systems. Consequently, these findings should be interpreted with caution, particularly regarding their generalizability to the broader turkey industry. Future investigations would benefit from incorporating sequences from a wider range of geographic areas, management systems, and production types. Such expanded sampling would not only improve the representativeness of the dataset but also enhance the ability to identify potential epidemiological patterns and emerging viral lineages on a global scale.

Amino acid-based phylogenies fundamentally reflect the changes in the protein sequence, and therefore detect non-synonymous mutations. This approach is particularly valuable when the goal is to infer potential functional or antigenic differences among viral strains such as virulence, host interaction, immune evasion, or replication efficiency. In contrast, synonymous mutations do not change the amino acid sequence and are therefore not reflected in amino acid–based phylogenetic trees, and such properties are especially helpful for tracking evolution (22, 55). In this study, our phylogenetic analyses were based on nucleotide sequences, which incorporate both synonymous and non-synonymous mutations. This approach provides a more comprehensive view of genetic variation among ARV strains and allows for the detection of evolutionary patterns that may be missed in amino acid–based analyses and offer insights into overall population structure and transmission dynamics with greater resolution. That being said, amino-acid-based classifications have the potential for phenotypic prediction; however, they may not fully capture genetic diversity. Therefore, it should also be noted that while the M2, σC, and L3 segments are immunologically important, the genotypes identified here cannot be directly translated into phenotypes, such as antigenicity or virulence as validating these traits requires a more thorough analysis and is beyond the scope of this study.

In this study, WGS was conducted on turkey ARV isolates that were cultured using a chicken cell line (LMH). This might potentially introduce a pre-analytic bias, as turkey-origin viruses may have undergone mutations to better adapt to the chicken-derived cells. In theory, such adaptations could involve phenotypic or genomic changes, particularly in viral attachment proteins or replication mechanisms, that enhance viral fitness in a non-native host. Although there are no studies specifically documenting this in turkey ARV isolates cultured with LMH cells, similar host-adaptive mutations have been observed in other viruses, particularly after repeated passaging in cell culture (56). While repeated passages were not performed in this study, the use of a chicken cell line still presents a possible source of bias. In this study, the workflow was streamlined: fresh samples were aseptically collected for both viral PCR and cell culture, and virus sequencing was performed only on samples that tested positive by both methods following the observation of CPEs. However, a potential limitation of the sequencing workflow is that not all ARVs produce readily observable CPEs (57). Previous studies have indicated that ARVs may not consistently induce CPEs in certain cell lines, such as quail myoblast clone 5 (QM5), and can be difficult to identify in LMH cells, as these cells tend to detach and float when infected with ARVs (11, 57). The plaque assay offers an alternative, particularly useful for QM5 cells due to their strong adherence and ability to form plaques (57). Genotyping classification is feasible only when high-quality sequence data are obtained. Therefore, careful attention to virus culture methods and sequencing procedures is essential to ensure reliable results.

In the clustering performance evaluation, weaker silhouette scores were primarily attributed to a limited number of sequences sharing similar phylogenetic relationships. Consequently, these sequences were grouped with neighboring sequences of similar nucleotide similarity. Increasing the distance threshold to include more isolates within a single segment did not improve silhouette scores. This is because, given the limited cluster diversity in the phylogenetic space, higher distance thresholds incorporate more sequences into the same cluster, increasing heterogeneity in nucleotide sequence distances (39). As a result, using a higher threshold may lead to suboptimal clustering and weaken the inferred correlation with evolutionary relationships, particularly when the population size is low. However, with the inclusion of additional sequences and a more extended temporal evaluation in the future, the emergence of more distinct clusters is expected, and the classification may be more robust (58).

Our comparison of clustering methods establishes a foundation for the fine-scale classification of ARV, providing a classification for as many sequences as possible while addressing the needs of animal health professionals who use sequence data for disease monitoring and management. Despite advantages of this approach, there are still approximately 30% of isolates that did not form a distinct constellation pattern/genotype (defined as a major group where more than 4 isolates share the same constellation of M2–σC–L3). This could be attributed to several factors: (1) low prevalence of these isolates in the field, (2) insufficient sampling to fully represent the ARV genotype diversity, (3) limited sampling timeframe, (4) high frequency of reassortment and mutation, and (5) overly strict parameters within the clustering methods. In this typing scheme, the unique evolutionary trajectory of reassortment is sometimes evident; however, without considering metadata, such as bird/virus sources and the timing of infection/collection, establishing clear epidemiologic links for minor groups is challenging, and assigning these groups to specific genotypes is difficult. To address this, continuous monitoring, followed by sequencing of ARV isolates from the field, could help expand and strengthen the current dataset and enhance the typing scheme.

It should be noted that autogenous vaccines against ARV have been widely employed in the US commercial turkey industry within the last decade due to the unavailability of commercial vaccines against emerging and diverse field strains (59). This novel typing scheme has the potential to improve vaccine selection by enabling targeted protection against predominant strains identified through genotyping. However, it is also important to recognize that the use of autogenous vaccines may also impose strong immune selection pressure on the viral population, potentially influencing viral evolution. When multiple genetic variants co-circulate, such pressure can favor the survival and spread of escape variants, particularly those with mutations in antigenic regions that allow them to evade vaccine-induced immune responses. Notably, co-circulation of ARV strains in the field appears to be common (49, 60) To address this, ongoing monitoring of field virus populations using WGS, in conjunction with the genotyping scheme could support the more precise formulation of autogenous vaccines. Such an approach would improve the match between vaccine composition and co-circulating strains and allow for more strategic vaccine deployment to anticipate and mitigate immune escape dynamics.

Both horizontal and vertical transmission of ARV have been demonstrated experimentally. Additionally, ARV is part of the normal virome in multiple avian species, which allows for cross species spillover events as well as the segmented nature of this virus leading to reassortment events well documented in this study and others. All these factors create a complex ARV ecology of infection and makes it reconstruct their genetic makeup for epidemiologic tracking purposes. However, the approach presented in this study provides the first steps in addresses this gap constitutes a useful tool for epidemiologic investigations of ARV transmission in the field using genetic information. This scheme found that multiple meat-type farms or farms with parent-progeny relationships sharing the same genotype likely represent a specific ARV transmission event and most likely linked to the bird source. Specifically, several cases of vertical transmission were suspected in Genotypes II, III, and VI, which showed direct evidence of the same genotypes in both meat-type farms and source birds at parent sites (II and III), or from grandparent to parent sites (VI). The remaining genotypes (I, IV, V, VII, and VIII) likely indicate a common source of the virus, with transmission occurring vertically (through common shared bird sources; I, IV, V, and VIII) and/or horizontally (within the same farm operations; I, IV, and VII). Although we employed an unbiased and rigorous methodology, bias may still be inherent in this analysis as data shared by breeder companies may be limited. Additionally, other transmission routes, such as those involving shared personnel, equipment, and transportation, are more difficult to characterize due to challenges in obtaining relevant data.

## 5 Conclusion

The genotyping with methodologies for clustering and grouping segmented viruses based on phylogeny, with a robust comparison of different methods, provides valuable insights for ARV epidemiology studies and lays the foundation for fine-scale classification of ARV in turkeys. The next steps to build upon this work involve testing the performance and robustness of this genotyping method on an ongoing basis. This will assess the ability of the classification system to accommodate the expanding genetic diversity as the virus continues to evolve. Additional steps include developing tools for rapid labeling of genetic variants, streamlining genotyping procedures for future implementation and potentially adopting this scheme for ARV in chickens and other poultry species to support the entirety of the poultry industry. Finally, this genotyping system can be a basis upon which other classifications, antigenic (serotype) or pathotype prediction, could be added. These efforts aim to ensure that the ARV typing systems meets the needs of diagnostic laboratories and animal health professionals in a timely manner, ultimately helping to better control the disease.

## Data Availability

The datasets presented in this study can be found in online repositories. The names of the repository/repositories and accession number(s) can be found at: https://www.ncbi.nlm.nih.gov/genbank/ under the accession numbers PV405945 - PV406034 (L1 gene), PV406035 - PV406122 (L2 gene), PV406123 - PV406210 (L3 gene), PV406211 - PV406300 (M1 gene), PV406301 - PV406392 (M2 gene), PV406393 - PV406484 (M3 gene), PV406485 - PV406575 (S1 gene), PV406576 - PV406664 (S2 gene), PV406665 - PV406757 (S3 gene), PV406758 - PV406846 (S4 gene).

## Footnotes

^1^ http://tree.bio.ed.ac.uk/software/figtree/

## Supplementary material

SUPPLEMENTARY FIGURE S1 Silhouette score comparison of sequence distances for L segments using TreeCluster under distance thresholds of 0.035 and 0.045.

SUPPLEMENTARY FIGURE S2 Silhouette score comparison of sequence distances for M segments using TreeCluster under distance thresholds of 0.035 and 0.045.

SUPPLEMENTARY FIGURE S3 Silhouette score comparison of sequence distances for S segments using TreeCluster under distance thresholds of 0.035 and 0.045.

SUPPLEMENTARY FIGURE S4 Maximum-likelihood phylogenetic tree of the L1 gene **(a)**, L2 **(b)**, and L3 **(c)**, with branches and tips colored by assigned cluster number ('-1' indicates singletons) under distance thresholds of 0.035.

SUPPLEMENTARY FIGURE S5 Maximum-likelihood phylogenetic tree of the M1 gene **(a)**, M2 **(b)**, and M3 **(c)**, with branches and tips colored by assigned cluster number ('-1' indicates singletons) under distance thresholds of 0.035.

SUPPLEMENTARY FIGURE S6 Maximum-likelihood phylogenetic tree of the σC gene **(a)**, S2 **(b)**, S3 **(c)**, and S4 **(d)** with branches and tips colored by assigned cluster number ('-1' indicates singletons) under distance thresholds of 0.035.

SUPPLEMENTARY FIGURE S7 Heat map of average pairwise nucleotide similarity within (highlighted) and between clusters of L segments.

SUPPLEMENTARY FIGURE S8 Heat map of average pairwise nucleotide similarity within (highlighted) and between clusters of M segments.

SUPPLEMENTARY FIGURE S9 Heat map of average pairwise nucleotide similarity within (highlighted) and between clusters of S segments.

SUPPLEMENTARY TABLE S1 Metadata from Iowa State University Veterinary Diagnostic Laboratory (ISU-VDL) and industry collaborators.

SUPPLEMENTARY TABLE S2 Best-fitting nucleotide substitution models for turkey avian reovirus genomic segments determined by Bayesian Information Criterion (BIC) scores.

## References

[1] Pitcovski J Goyal SM. Avian reovirus infections In: Swayne DE, editor. Diseases of poultry. 14th ed. Hoboken, USA: John Wiley & Sons, Inc. (2020). 382–400. doi: 10.1002/9781119371199.ch11

[2] French D. Incidence and economic impact of reovirus in the poultry Industries in the United States. Avian Dis. (2022) 66:432–4. doi: 10.1637/aviandiseases-D-22-99993, PMID: 36715475

[3] Gallardo RA. Molecular characterization of variant avian reoviruses and their relationship with antigenicity and pathogenicity. Avian Dis. (2022) 66:443–6. doi: 10.1637/aviandiseases-D-22-99995, PMID: 36715477

[4] Markis M. Evaluation of pathogenicity and antigenicity of avian reoviruses and disease control through vaccination. Avian Dis. (2022) 66:435–42. doi: 10.1637/aviandiseases-D-22-99994, PMID: 36715476

[5] Ni Y Kemp MC. A comparative study of avian reovirus pathogenicity: virus spread and replication and induction of lesions. Avian Dis. (1995) 39:554. doi: 10.2307/1591809, PMID: 8561741

[6] Egaña-Labrin S Jerry C Roh H Da Silva A Corsiglia C Crossley B. Avian reoviruses of the same genotype induce different pathology in chickens. Avian Dis. (2021) 65:530–40. doi: 10.1637/0005-2086-65.4.530, PMID: 35068095

[7] Kant A Balk F Born L van Roozelaar D Heijmans J Gielkens A. Classification of Dutch and German avian reoviruses by sequencing the σ C protein. Vet Res. (2003) 34:203–12. doi: 10.1051/vetres:2002067 12657212

[8] Mor SK Verma H Sharafeldin TA Porter RE Jindal N Ziegler A. Characterization of S class gene segments of a newly isolated Turkey arthritis reovirus. Virology. (2014) 464-465:33–44. doi: 10.1016/j.virol.2014.06.009, PMID: 25043587

[9] Mor SK Marthaler D Verma H Sharafeldin TA Jindal N Porter RE. Phylogenetic analysis, genomic diversity and classification of M class gene segments of Turkey reoviruses. Vet Microbiol. (2015) 176:70–82. doi: 10.1016/j.vetmic.2015.01.005, PMID: 25655814

[10] Mor SK Sharafeldin TA Porter RE Goyal SM. Molecular characterization of L class genome segments of a newly isolated Turkey arthritis reovirus. Infect Genet Evol. (2014) 27:193–201. doi: 10.1016/j.meegid.2014.07.012, PMID: 25057811

[11] Lu H Tang Y Dunn PA Wallner-Pendleton EA Lin L Knoll EA. Isolation and molecular characterization of newly emerging avian reovirus variants and novel strains in Pennsylvania, USA, 2011–2014. Sci Rep. (2015) 5:1–11. doi: 10.1038/srep14727, PMID: 26469681 PMC4606735

[12] Gál B Varga-Kugler R Ihász K Kaszab E Farkas S Marton S. A snapshot on the genomic epidemiology of Turkey reovirus infections, Hungary. Animals. (2023) 13:3504. doi: 10.3390/ani13223504, PMID: 38003122 PMC10668827

[13] Hsueh C-S Zeller M Hashish A Fasina O Piñeyro P Li G. Investigation of avian reovirus evolution and cross-species transmission in Turkey hosts by segment-based temporal analysis. Viruses. (2025) 17:926. doi: 10.3390/v17070926, PMID: 40733544 PMC12300447

[14] Sellers HS. Avian reoviruses from clinical cases of tenosynovitis: an overview of diagnostic approaches and 10-year review of isolations and genetic characterization. Avian Dis. (2022) 66:420–6. doi: 10.1637/aviandiseases-D-22-99990, PMID: 36715473

[15] Arafa E Abdien HM Zain El-Abideen MA Diab E Tarek M El-Dimerdash MM. A new record of avian reovirus genogroup clusters isolated and molecularly characterized in chickens in Egypt. Beni-Suef Univ J Basic Appl Sci. (2024) 13:122. doi: 10.1186/s43088-024-00568-9

[16] Rafique S Rashid F Wei Y Zeng T Xie L Xie Z. Avian orthoreoviruses: a systematic review of their distribution, dissemination patterns, and genotypic clustering. Viruses. (2024) 16:1056. doi: 10.3390/v16071056, PMID: 39066218 PMC11281703

[17] Ma X Li W Liu Z Zuo Z Dang X Gao H. Isolation, identification, and pathogenicity of an avian reovirus epidemic strain in Xinjiang, China. Viruses. (2025) 17:499. doi: 10.3390/v17040499, PMID: 40284942 PMC12031336

[18] Youk S Torchetti MK Lantz K Lenoch JB Killian ML Leyson C. H5N1 highly pathogenic avian influenza clade 2.3.4.4 b in wild and domestic birds: introductions into the United States and reassortments, December 2021–April 2022. Virology. (2023) 587:109860. doi: 10.1016/j.virol.2023.109860 37572517

[19] Group EW. Toward a unified nomenclature system for highly pathogenic avian influenza virus (H5N1). Emerg Infect Dis. (2008) 14:e1. doi: 10.3201/eid1407.071681, PMID: 18598616 PMC2600337

[20] Matthijnssens J Heylen E Zeller M Rahman M Lemey P Van Ranst M. Phylodynamic analyses of rotavirus genotypes G9 and G12 underscore their potential for swift global spread. Mol Biol Evol. (2010) 27:2431–6. doi: 10.1093/molbev/msq137, PMID: 20522727

[21] Matthijnssens J Ciarlet M Rahman M Attoui H Bányai K Estes MK. Recommendations for the classification of group a rotaviruses using all 11 genomic RNA segments. Arch Virol. (2008) 153:1621–9. doi: 10.1007/s00705-008-0155-1, PMID: 18604469 PMC2556306

[22] Matthijnssens J Ciarlet M Heiman E Arijs I Delbeke T McDonald SM. Full genome-based classification of rotaviruses reveals a common origin between human Wa-like and porcine rotavirus strains and human DS-1-like and bovine rotavirus strains. J Virol. (2008) 82:3204–19. doi: 10.1128/JVI.02257-07, PMID: 18216098 PMC2268446

[23] Pérez-Losada M Arenas M Galán JC Palero F González-Candelas F. Recombination in viruses: mechanisms, methods of study, and evolutionary consequences. Infect Genet Evol. (2015) 30:296–307. doi: 10.1016/j.meegid.2014.12.022, PMID: 25541518 PMC7106159

[24] Rabadan R Levine AJ Krasnitz M. Non-random reassortment in human influenza a viruses. Influenza Other Respir Viruses. (2008) 2:9–22. doi: 10.1111/j.1750-2659.2007.00030.x, PMID: 19453489 PMC4634327

[25] Nibert ML Margraf RL Coombs KM. Nonrandom segregation of parental alleles in reovirus reassortants. J Virol. (1996) 70:7295–300. doi: 10.1128/jvi.70.10.7295-7300.1996, PMID: 8794386 PMC190792

[26] Egaña-Labrin S Hauck R Figueroa A Stoute S Shivaprasad H Crispo M. Genotypic characterization of emerging avian reovirus genetic variants in California. Sci Rep. (2019) 9:1–10. doi: 10.1038/s41598-019-45494-4 31249323 PMC6597705

[27] Dawe W Kapczynski D Linnemann E Gauthiersloan V Sellers H. Analysis of the immune response and identification of antibody epitopes against the sigma C protein of avian Orthoreovirus following immunization with live or inactivated vaccines. Avian Dis. (2022) 66:465–78. doi: 10.1637/aviandiseases-D-22-99992, PMID: 36715481

[28] Katoh K Standley DM. A simple method to control over-alignment in the MAFFT multiple sequence alignment program. Bioinformatics. (2016) 32:1933–42. doi: 10.1093/bioinformatics/btw108, PMID: 27153688 PMC4920119

[29] Nguyen L-T Schmidt HA Von Haeseler A Minh BQ. IQ-TREE: a fast and effective stochastic algorithm for estimating maximum-likelihood phylogenies. Mol Biol Evol. (2015) 32:268–74. doi: 10.1093/molbev/msu300, PMID: 25371430 PMC4271533

[30] Kalyaanamoorthy S Minh BQ Wong TK Von Haeseler A Jermiin LS. Model finder: fast model selection for accurate phylogenetic estimates. Nat Methods. (2017) 14:587–9. doi: 10.1038/nmeth.4285 28481363 PMC5453245

[31] Hoang DT Chernomor O Von Haeseler A Minh BQ Vinh LS. UFBoot2: improving the ultrafast bootstrap approximation. Mol Biol Evol. (2018) 35:518–22. doi: 10.1093/molbev/msx281, PMID: 29077904 PMC5850222

[32] Tang Y Lu H. Whole genome alignment based one-step real-time RT-PCR for universal detection of avian orthoreoviruses of chicken, pheasant and Turkey origins. Infect Genet Evol. (2016) 39:120–6. doi: 10.1016/j.meegid.2016.01.018, PMID: 26812128

[33] Shen H Zhang J Gauger PC Burrough ER Zhang J Harmon K. Genetic characterization of porcine sapoviruses identified from pigs during a diarrhoea outbreak in Iowa, 2019. Transbound Emerg Dis. (2022) 69:1246–55. doi: 10.1111/tbed.14087, PMID: 33780163

[34] Bolger AM Lohse M Usadel B. Trimmomatic: a flexible trimmer for Illumina sequence data. Bioinformatics. (2014) 30:2114–20. doi: 10.1093/bioinformatics/btu170, PMID: 24695404 PMC4103590

[35] Danecek P Bonfield JK Liddle J Marshall J Ohan V Pollard MO. Twelve years of SAMtools and BCFtools. Gigascience. (2021) 10:giab008. doi: 10.1093/gigascience/giab008, PMID: 33590861 PMC7931819

[36] Simpson JT Wong K Jackman SD Schein JE Jones SJ Birol I. ABySS: a parallel assembler for short read sequence data. Genome Res. (2009) 19:1117–23. doi: 10.1101/gr.089532.108, PMID: 19251739 PMC2694472

[37] Bankevich A Nurk S Antipov D Gurevich AA Dvorkin M Kulikov AS. SPAdes: a new genome assembly algorithm and its applications to single-cell sequencing. J Comput Biol. (2012) 19:455–77. doi: 10.1089/cmb.2012.0021, PMID: 22506599 PMC3342519

[38] Robinson JT Thorvaldsdóttir H Winckler W Guttman M Lander ES Getz G. Integrative genomics viewer. Nat Biotechnol. (2011) 29:24–6. doi: 10.1038/nbt.1754, PMID: 21221095 PMC3346182

[39] Balaban M Moshiri N Mai U Jia X Mirarab S. Tree cluster: clustering biological sequences using phylogenetic trees. PLoS One. (2019) 14:e0221068. doi: 10.1371/journal.pone.0221068, PMID: 31437182 PMC6705769

[40] Zhang J. (2019) Multidimensional scaling for phylogenetics. Burnaby, British Columbia: Simon Fraser University, Department of Statistics and Actuarial Science.

[41] Dalmaijer ES Nord CL Astle DE. Statistical power for cluster analysis. BMC Bioinformatics. (2022) 23:205. doi: 10.1186/s12859-022-04675-1, PMID: 35641905 PMC9158113

[42] Ayalew LE Ahmed KA Mekuria ZH Lockerbie B Popowich S Tikoo SK. The dynamics of molecular evolution of emerging avian reoviruses through accumulation of point mutations and genetic re-assortment. Virus evolution. (2020) 6:veaa025. doi: 10.1093/ve/veaa025, PMID: 32411390 PMC7211400

[43] Letunic I Bork P. Interactive tree of life (iTOL) v6: recent updates to the phylogenetic tree display and annotation tool. Nucleic Acids Res. (2024) 52:W78. doi: 10.1093/nar/gkae268, PMID: 38613393 PMC11223838

[44] Farkas SL Varga-Kugler R Ihász K Marton S Gál J Palya V. Genomic characterization of avian and neoavian orthoreoviruses detected in pheasants. Virus Res. (2021) 297:198349. doi: 10.1016/j.virusres.2021.198349, PMID: 33631220

[45] Farkas SL Marton S Dandár E Kugler R Gál B Jakab F. Lineage diversification, homo- and heterologous reassortment and recombination shape the evolution of chicken orthoreoviruses. Sci Rep. (2016) 6:1–9. doi: 10.1038/srep36960 27830770 PMC5103266

[46] Pantin-Jackwood M Spackman E Day J. Pathology and virus tissue distribution of Turkey origin reoviruses in experimentally infected Turkey poults. Vet Pathol. (2007) 44:185–95. doi: 10.1354/vp.44-2-185, PMID: 17317795

[47] Menendez N Calnek B Cowen B. Experimental egg-transmission of avian reovirus. Avian Dis. (1975) 19:104–11. doi: 10.2307/1588960, PMID: 164174

[48] Jones R Onunkwo O. Studies on experimental tenosynovitis in light hybrid chickens. Avian Pathol. (1978) 7:171–81. doi: 10.1080/03079457808418268, PMID: 18770368

[49] Liu HJ Lee LH Hsu HW Kuo LC Liao MH. Molecular evolution of avian reovirus: evidence for genetic diversity and reassortment of the S-class genome segments and multiple cocirculating lineages. Virology. (2003) 314:336–49. doi: 10.1016/s0042-6822(03)00415-x, PMID: 14517086

[50] De Carli S Wolf JM Gräf T Lehmann FK Fonseca AS Canal CW. Genotypic characterization and molecular evolution of avian reovirus in poultry flocks from Brazil. Avian Pathol. (2020) 49:611–20. doi: 10.1080/03079457.2020.1804528 32746617

[51] Neumann G Green MA Macken CA. Evolution of highly pathogenic avian H5N1 influenza viruses and the emergence of dominant variants. J Gen Virol. (2010) 91:1984–95. doi: 10.1099/vir.0.020750-0, PMID: 20392897

[52] Spruit CM Zhu X Tomris I Ríos-Carrasco M Han AX Broszeit F. N-glycolylneuraminic acid binding of avian and equine H7 influenza a viruses. J Virol. (2022) 96:e02120–1. doi: 10.1128/jvi.02120-21, PMID: 35044215 PMC8906439

[53] Hu T Chitnis N Monos D Dinh A. Next-generation sequencing technologies: an overview. Hum Immunol. (2021) 82:801–11. doi: 10.1016/j.humimm.2021.02.012, PMID: 33745759

[54] Tucker T Marra M Friedman JM. Massively parallel sequencing: the next big thing in genetic medicine. Am J Hum Genet. (2009) 85:142–54. doi: 10.1016/j.ajhg.2009.06.022, PMID: 19679224 PMC2725244

[55] Peck KM Lauring AS. Complexities of viral mutation rates. J Virol. (2018) 92:10.1128/jvi. 01031-17. doi: 10.1128/JVI.01031-17, PMID: 29720522 PMC6026756

[56] Petersen H Matrosovich M Pleschka S Rautenschlein S. Replication and adaptive mutations of low pathogenic avian influenza viruses in tracheal organ cultures of different avian species. PLoS One. (2012) 7:e42260. doi: 10.1371/journal.pone.0042260, PMID: 22912693 PMC3418272

[57] Harrell TL Alvarez-Narvaez S Conrad SJ. Determination of a suitable avian cell line for the quantification of avian reovirus via plaque assays. Avian Dis. (2025) 69:212–6. doi: 10.1637/aviandiseases-D-24-00100, PMID: 40643941

[58] Smith MR. Robust analysis of phylogenetic tree space. Syst Biol. (2022) 71:1255–70. doi: 10.1093/sysbio/syab100, PMID: 34963003 PMC9366458

[59] Porter R, editor. (2018). Turkey reoviral arthritis update. Midwest poultry federation convention: Turkey health workshop. Minneapolis, Minnesota, USA, march 2018. Available online at:https://midwestpoultrycom/wp-content/uploads/2018/03/Porter-Robpdf Last accessed September; 2018.

[60] Jiang X Wei F He D Niu X Wu B Wu Q. Co-circulation of multiple genotypes of ARV in poultry in Anhui, China. Avian Pathol. (2023) 52:389–400. doi: 10.1080/03079457.2023.2226081, PMID: 37314823

