# Supplementary figures and images for: Constellation-based classification of avian reovirus in turkeys reveals shared virus origins among different meat-type farms
Source: Front Vet Sci. 2025 Sep 10;12:1648247. doi: 10.3389/fvets.2025.1648247 (PMC12457133; doi:10.3389/fvets.2025.1648247)

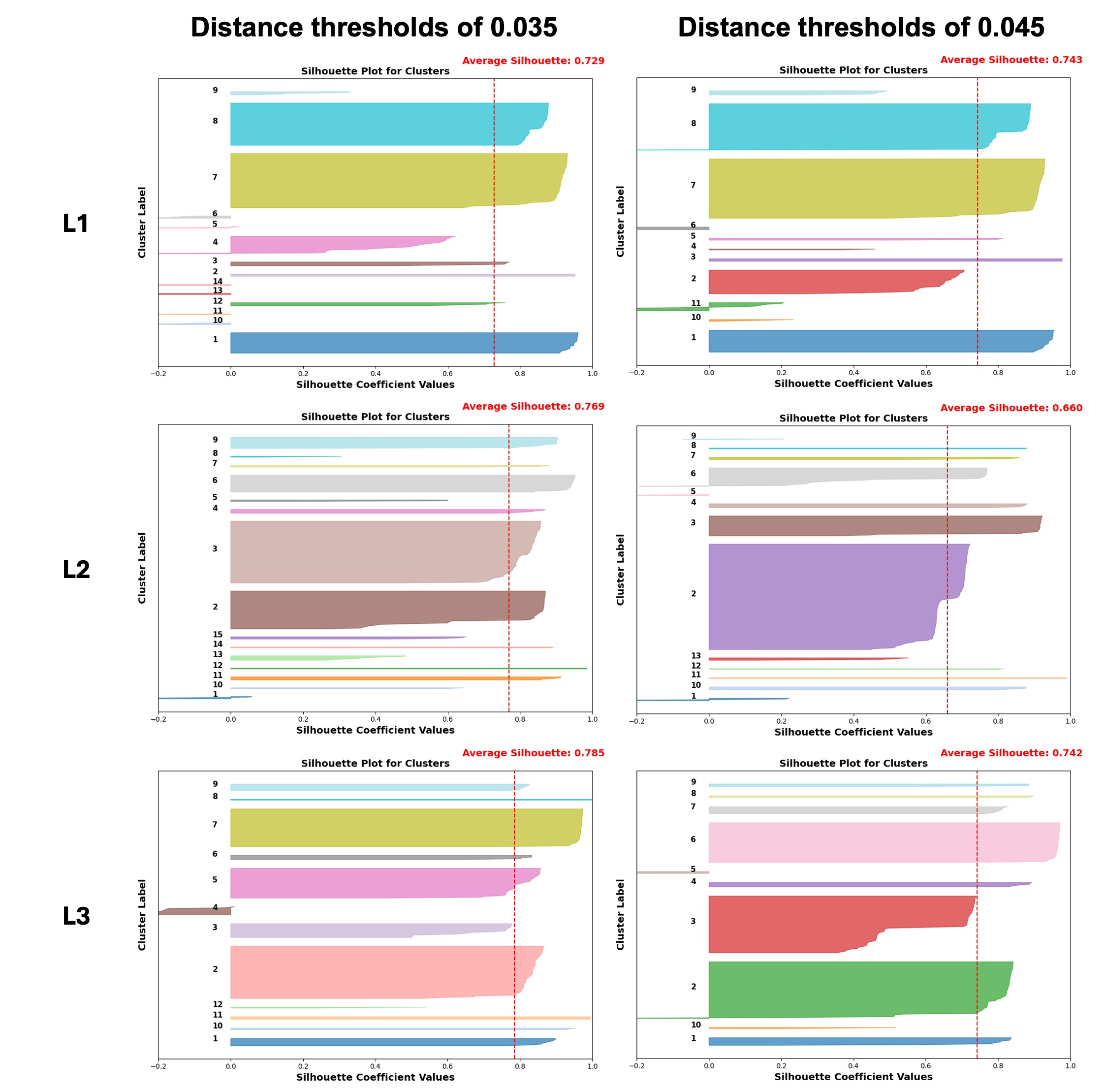

Supplement: SUPPLEMENTARY FIGURE S1 — Silhouette score comparison of sequence distances for L segments using TreeCluster under distance thresholds of 0.035 and 0.045. [file Image_1.JPEG]

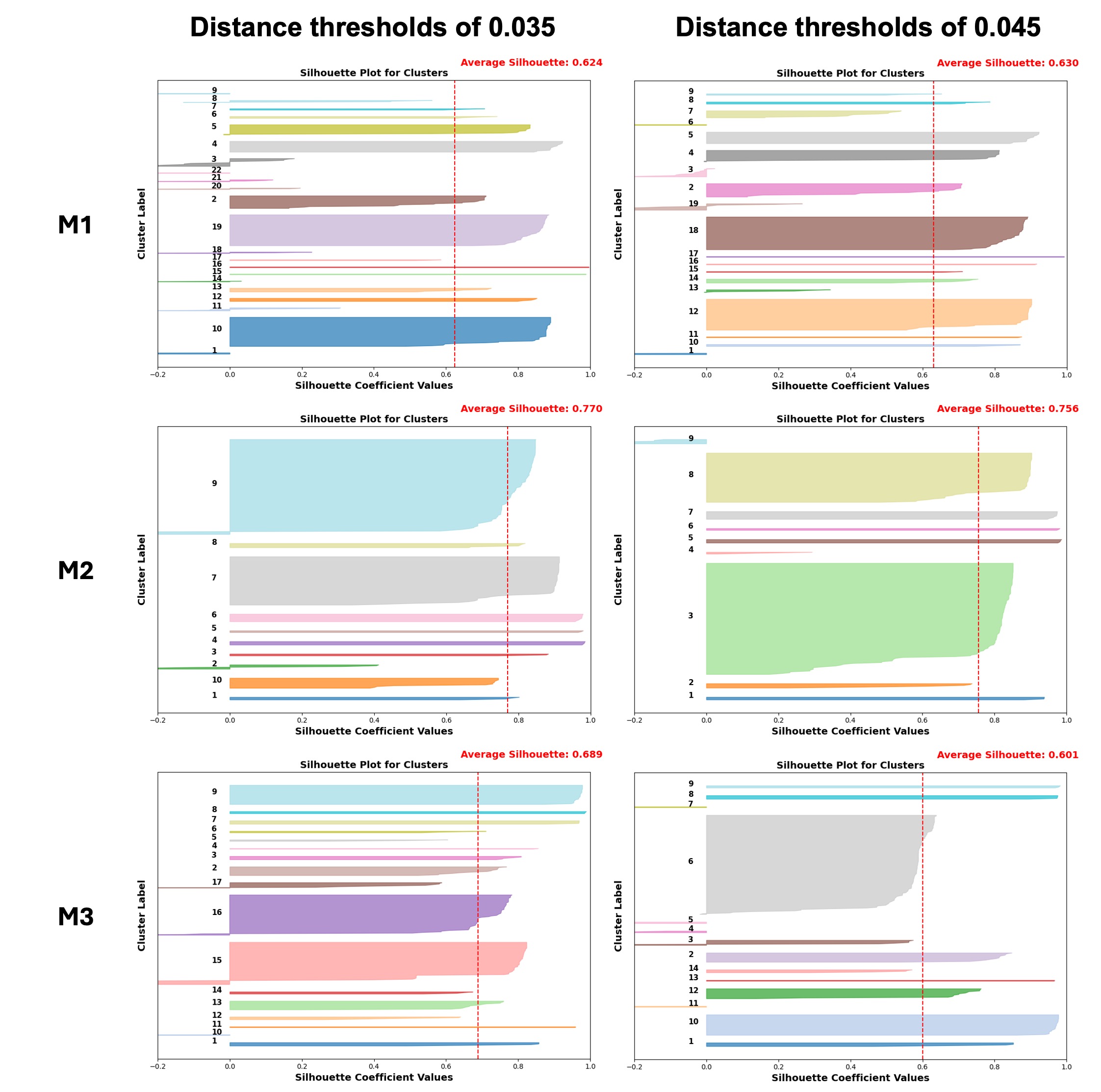

Supplement: SUPPLEMENTARY FIGURE S2 — Silhouette score comparison of sequence distances for M segments using TreeCluster under distance thresholds of 0.035 and 0.045. [file Image_2.JPEG]

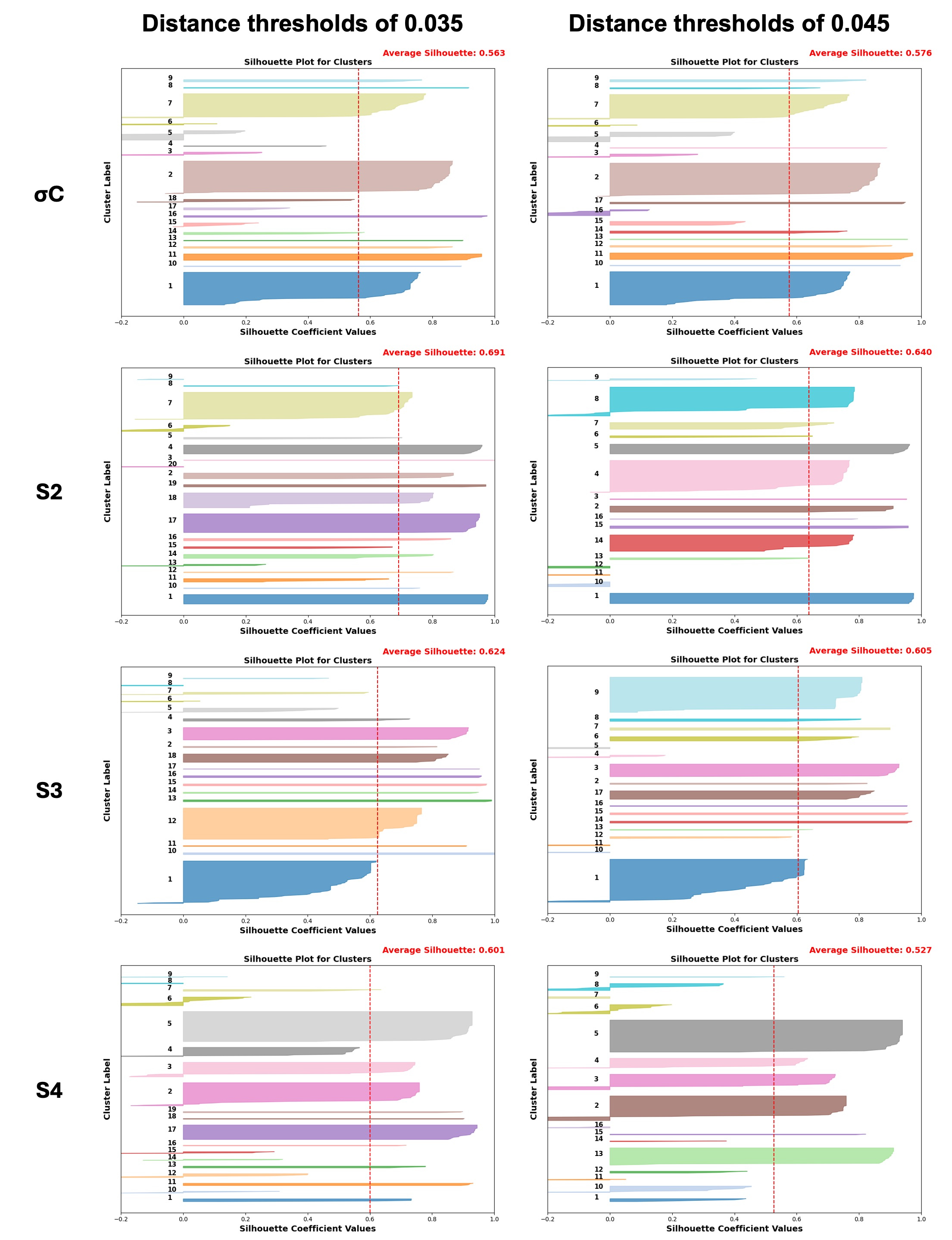

Supplement: SUPPLEMENTARY FIGURE S3 — Silhouette score comparison of sequence distances for S segments using TreeCluster under distance thresholds of 0.035 and 0.045. [file Image_3.JPEG]

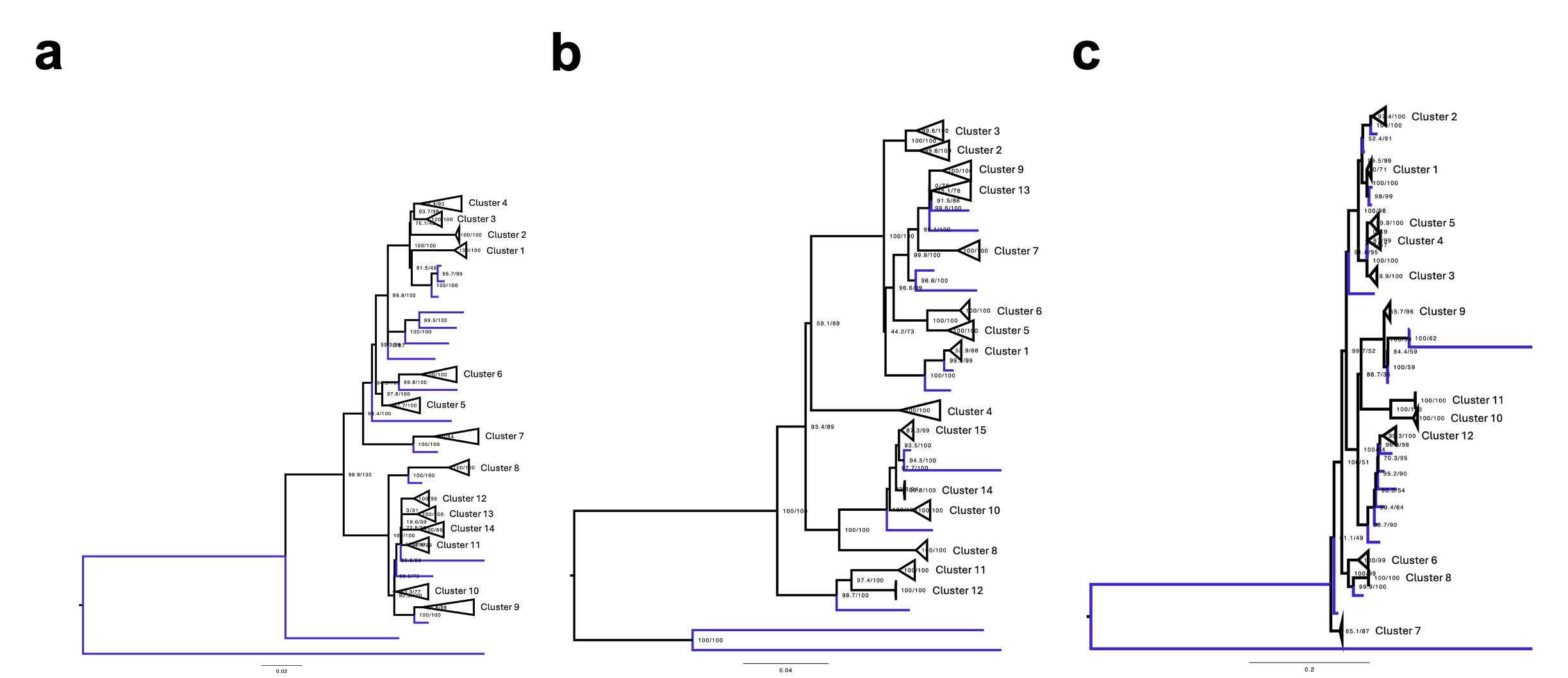

Supplement: SUPPLEMENTARY FIGURE S4 — Maximum-likelihood phylogenetic tree of the L1 gene (a), L2 (b), and L3 (c), with branches and tips colored by assigned cluster number ('-1' indicates singletons) under distance thresholds of 0.035. [file Image_4.JPEG]

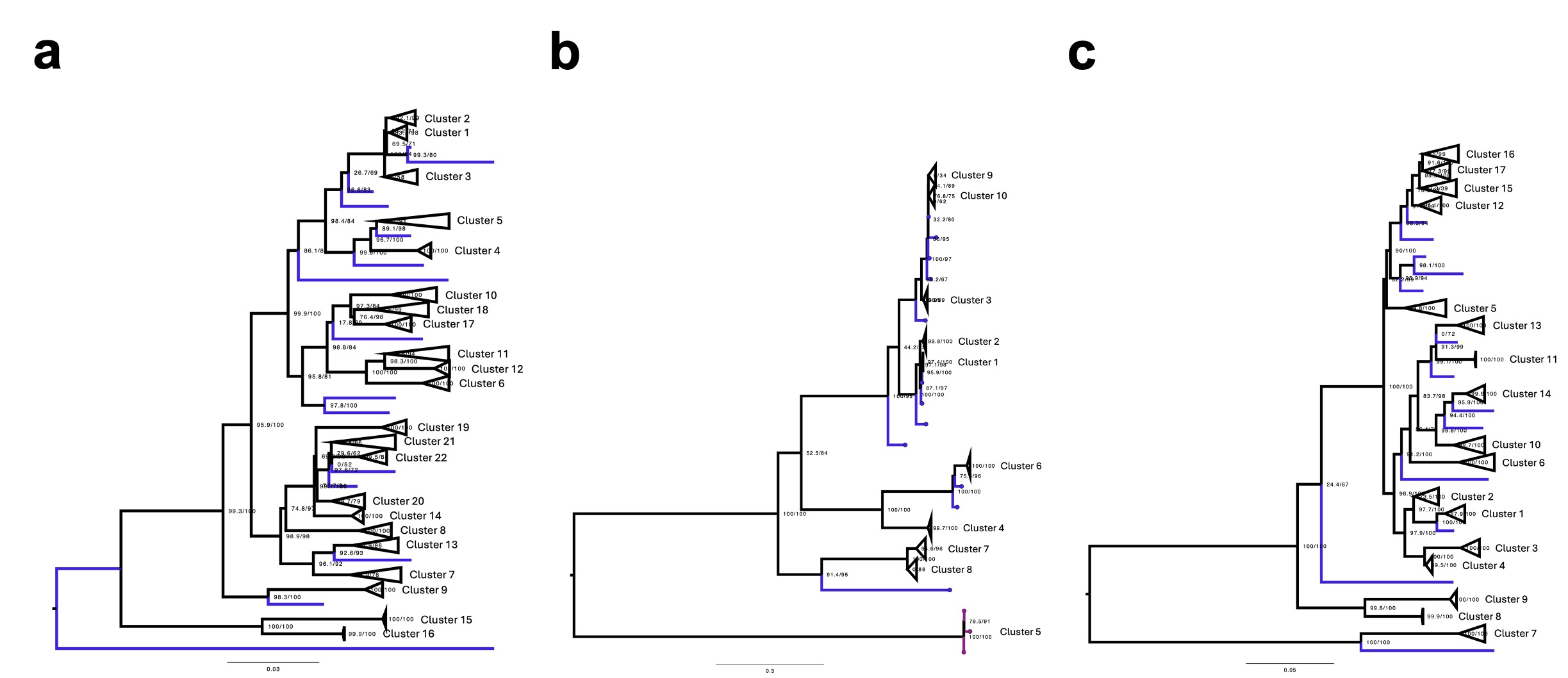

Supplement: SUPPLEMENTARY FIGURE S5 — Maximum-likelihood phylogenetic tree of the M1 gene (a), M2 (b), and M3 (c), with branches and tips colored by assigned cluster number ('-1' indicates singletons) under distance thresholds of 0.035. [file Image_5.JPEG]

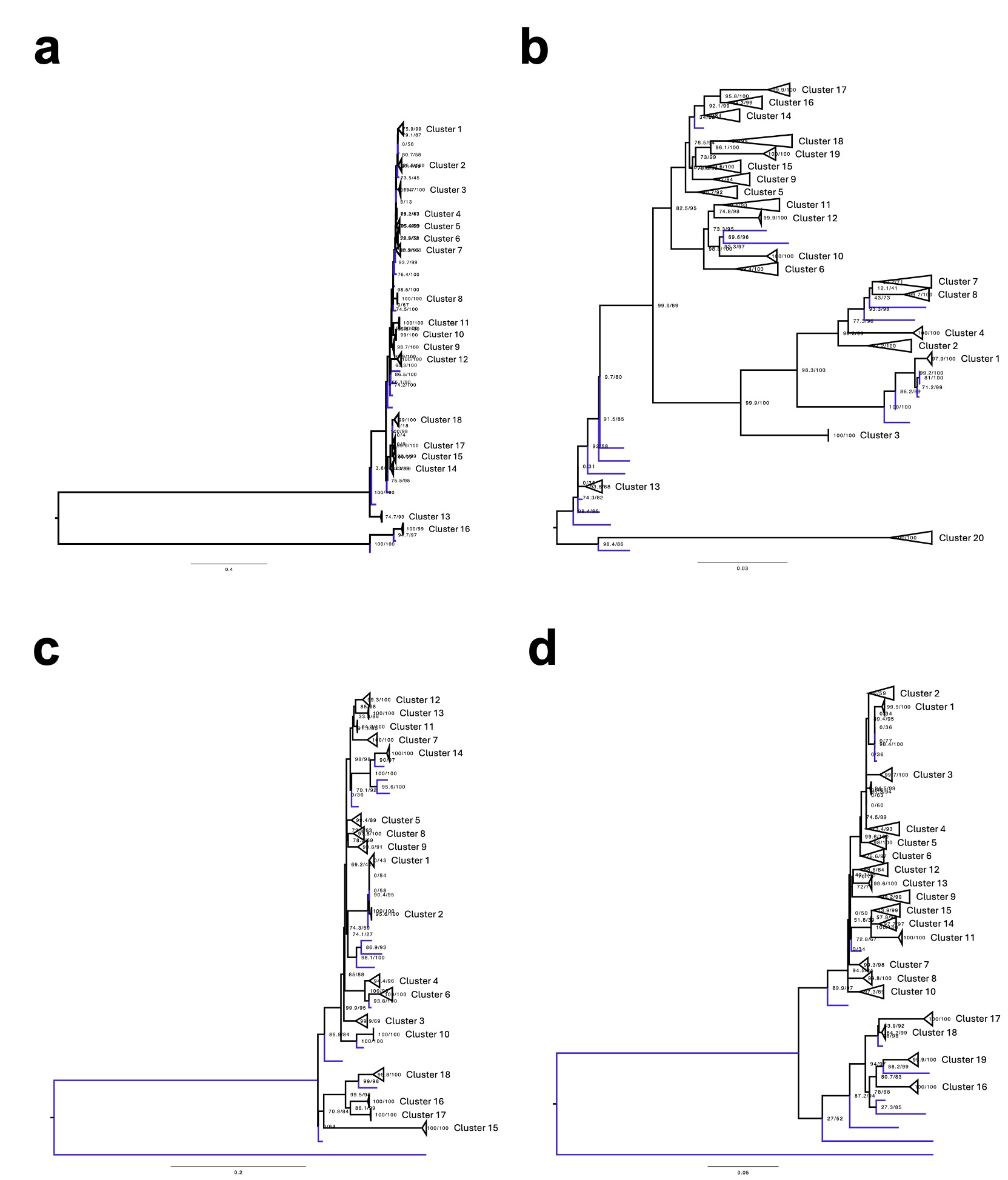

Supplement: SUPPLEMENTARY FIGURE S6 — Maximum-likelihood phylogenetic tree of the σC gene (a), S2 (b), S3 (c), and S4 (d) with branches and tips colored by assigned cluster number ('-1' indicates singletons) under distance thresholds of 0.035. [file Image_6.JPEG]

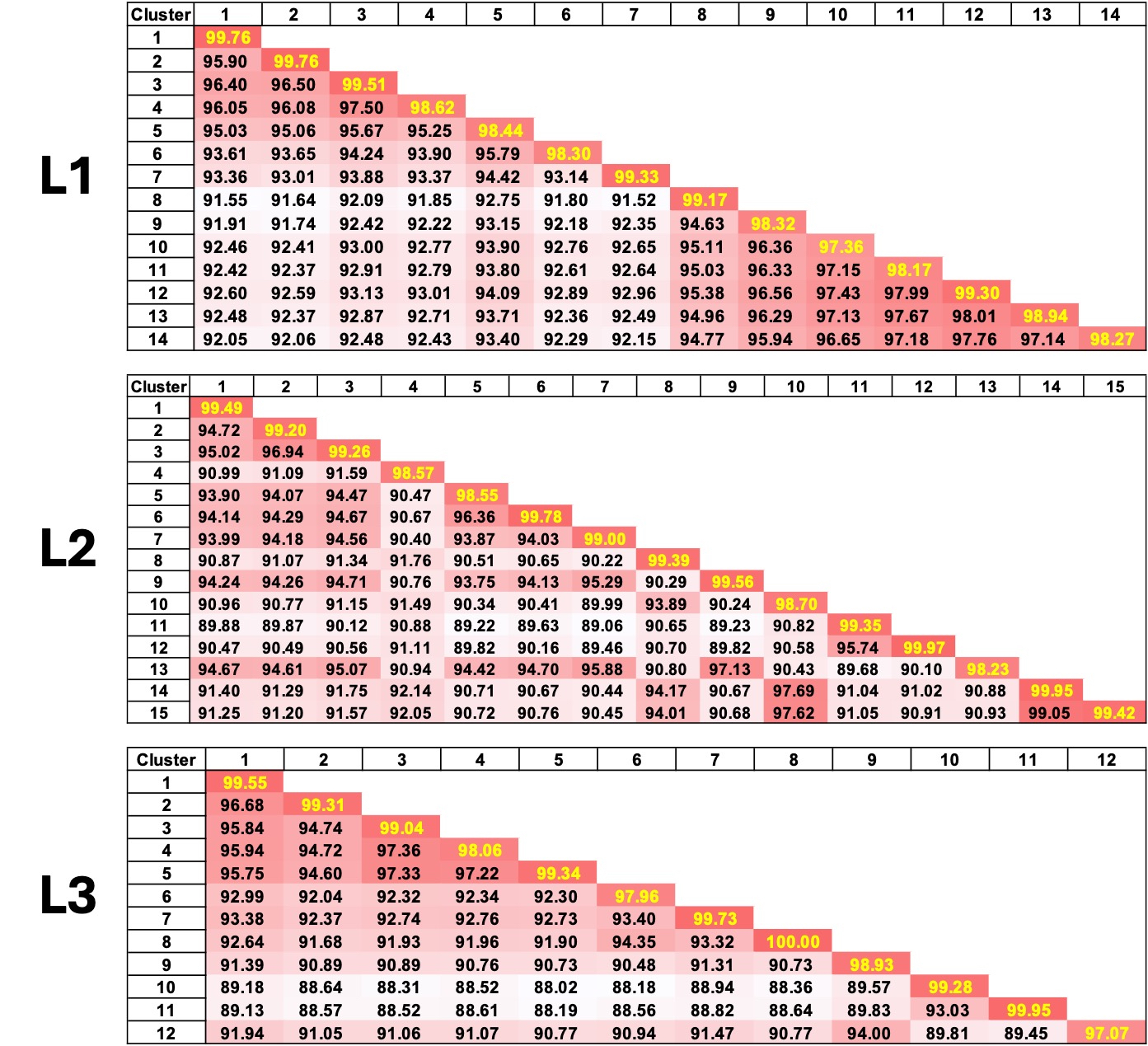

Supplement: SUPPLEMENTARY FIGURE S7 — Heat map of average pairwise nucleotide similarity within (highlighted) and between clusters of L segments. [file Image_7.JPEG]

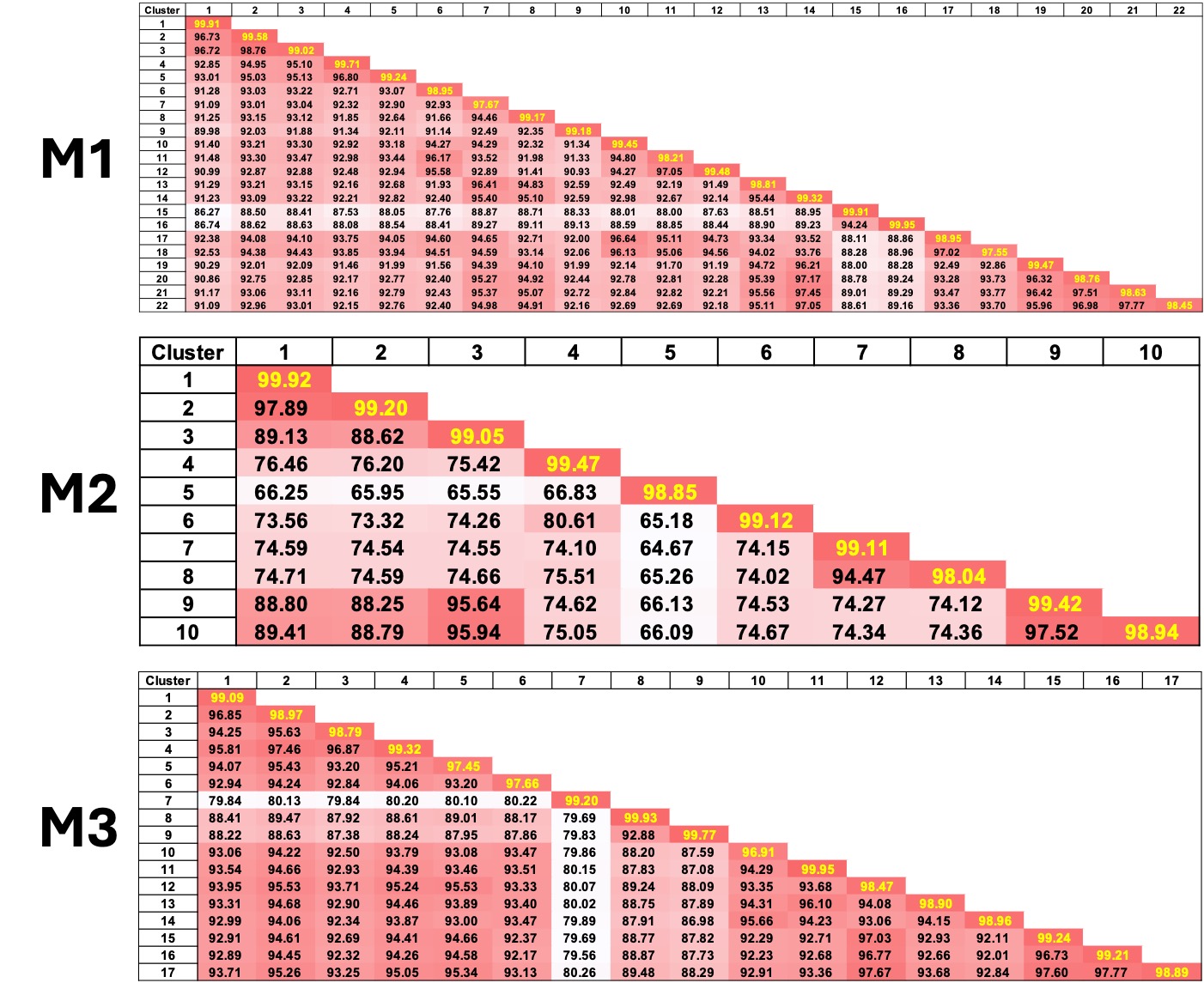

Supplement: SUPPLEMENTARY FIGURE S8 — Heat map of average pairwise nucleotide similarity within (highlighted) and between clusters of M segments. [file Image_8.JPEG]

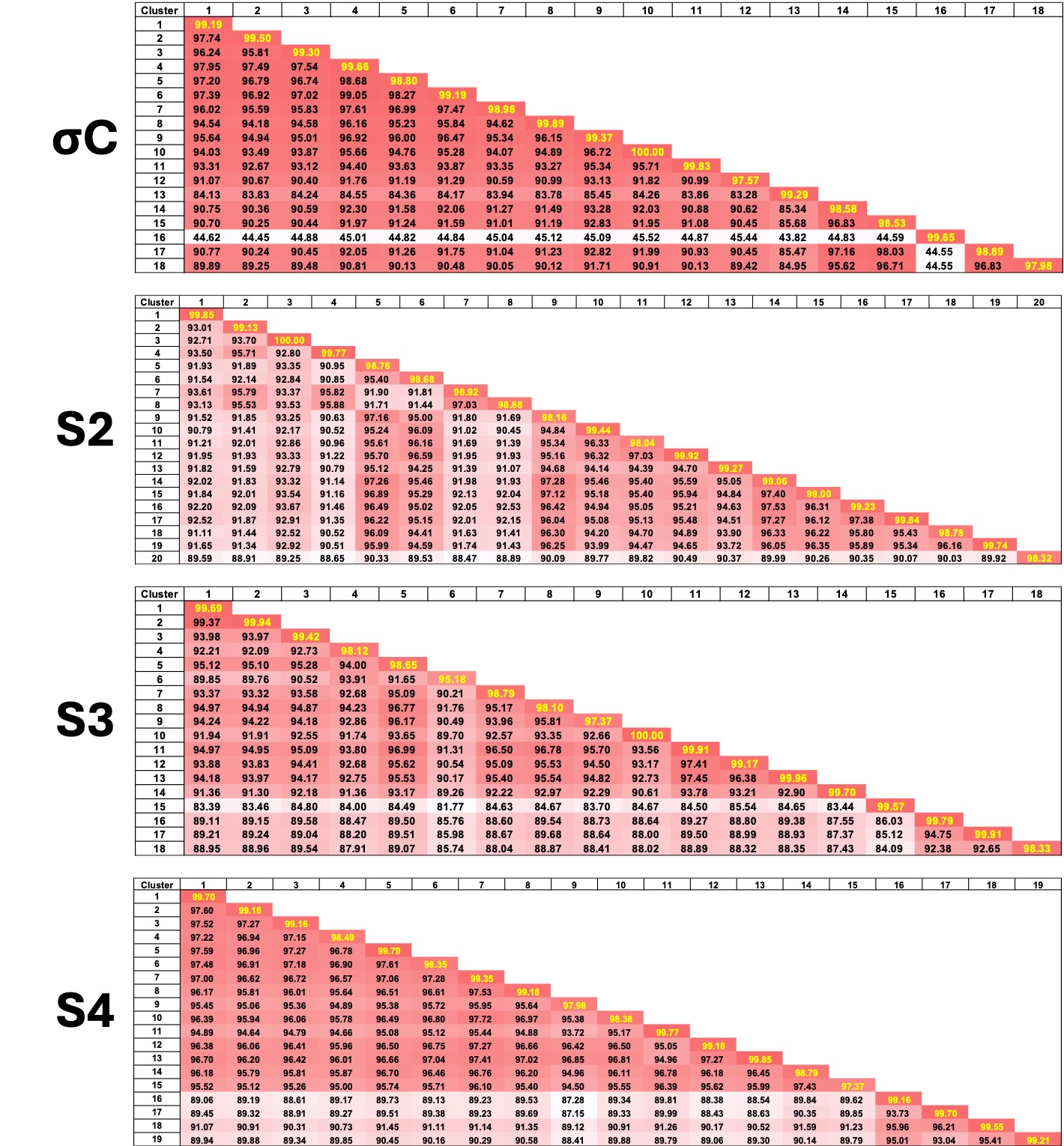

Supplement: SUPPLEMENTARY FIGURE S9 — Heat map of average pairwise nucleotide similarity within (highlighted) and between clusters of S segments. [file Image_9.JPEG]
