# Supplementary material for: Constellation-based classification of avian reovirus in turkeys reveals shared virus origins among different meat-type farms
Source: Front Vet Sci. 2025 Sep 10;12:1648247. doi: 10.3389/fvets.2025.1648247 (PMC12457133; doi:10.3389/fvets.2025.1648247)
Supplement: SUPPLEMENTARY TABLE S2 — Best-fitting nucleotide substitution models for turkey avian reovirus genomic segments determined by Bayesian Information Criterion (BIC) scores. [file Table_2.DOCX]

Supplemental Table 2. Best-fitting nucleotide substitution models for turkey avian reovirus genomic segments determined by Bayesian Information Criterion (BIC) scores.

|  | Best-fit model chosen according to BIC | BIC score |
| --- | --- | --- |
| L1 | K3Pu+F+I+G4 | 61485.110 |
| L2 | TVM+F+I+G4 | 60846.336 |
| L3 | TVM+F+I+G4 | 67298.149 |
| M1 | TVM+F+I+G4 | 42315.977 |
| M2 | TIM+F+I+G4 | 39761.903 |
| M3 | SYM+I+G4 | 37820.483 |
| σC | K3P+I+G4 | 24099.389 |
| S2 | TIMe+I+G4 | 21266.038 |
| S3 | TIMe+I+G4 | 21942.358 |
| S4 | K3Pu+F+G4 | 20049.995 |
